# Threshold effects of green technology application on sustainable grain production: Evidence from China

**DOI:** 10.3389/fpls.2023.1107970

**Published:** 2023-01-30

**Authors:** Jingdong Li, Qingning Lin

**Affiliations:** ^1^ Institute of Geographic Sciences and Natural Resources Research, Chinese Academy of Sciences, Beijing, China; ^2^ Key Laboratory of Regional Sustainable Development Modeling, Chinese Academy of Sciences, Beijing, China; ^3^ Institute of Agricultural Economics and Development, Chinese Academy of Agricultural Sciences, Beijing, China

**Keywords:** green technology, grain production, green total factor productivity, influence mechanism, threshold effects

## Abstract

Sustainable production is considered as an important approach to solve the dilemma of food insecurity. Green technologies have made contributions to improving food production and reducing environmental pollution. Studying the effects of green technologies on sustainable food production has great significance. The paper started with the influence mechanism of green technology application on the green total factor productivity of grain (GTFPG). With the GTFPG, green technology efficiency change of grain (GECG) and green technical progress change of grain (GTCG) measured, threshold models were constructed to explore the nonlinear impacts of various green technologies on GTFPG and the influence paths. Results indicated that the differences of GTFPG among provinces in China were decreased mainly due to the changes of GTCG, while the regional differences of GECG remained small. The impacts of green technologies had threshold effects that depended on the ecological effects of green technologies in different application stages, and were significantly different in the major and non-major grain producing areas. Meanwhile, significant differences existed in the influence paths of green technologies. In the major grain producing areas, green technologies were more likely to improve GTFPG through the GTCG path; while in the non-major grain producing areas, the GECG path and the GTCG path were both important to improve GTFPG. The differences of green technologies’ threshold effects and influence paths in the major and non-major grain producing areas were caused by regional technology preference, resource endowment and technology compatibility. This study emphasizes that the development of green technologies should fully consider the resource endowment and economic development of different regions, as well as the applicability and adoption rate of green technologies.

## 1 Introduction

Sustainable food security has been the foundation for global economic and social development. From Millennium Development Goals (MDGs) proposed by the United Nations to Sustainable Development Goals (SDGs), the focus of food security has shifted from extreme poverty and hunger to food security and nutrition improvement within the framework of sustainable agriculture (UN, 2001; UN, 2015; Clapp et al., 2022). However, shocks from pandemics, conflicts, natural disasters, climate change, and energy crisis have intensified the risk of global food insecurity (FSIN, 2022; Li and Song, 2022), further frustrating the progress of SDGs 1 and SDGs 2 (FAO et al., 2022).

In the complex natural, economic and social environment, sustainable production is considered as an important way out of food insecurity dilemma (Rahman et al., 2021; ECOSOC, 2022). Extensive management of traditional agriculture features high resource input and high energy consumption (Jin et al., 2012; Nagothu, 2018). Its pursuit of maximized output has brought about resource waste, land overdraft, non-point source pollution, greenhouse gas emissions, etc. (Shen et al., 2018; Li and Lin, 2022), especially in Asia, where extensive agricultural production mode is expected to increase greenhouse gas emissions by 37% in 2050 (Frank et al., 2019). Sustainable agriculture highlights the use of advanced technologies and management to ensure the quality of agricultural products and ecological security, and improve comprehensive economic benefits (Liu et al., 2020; Guo et al., 2022a). It takes into accounts economy, society and environment (Elkington, 1994; Purvis et al., 2019), and strikes a balance between agricultural development and environmental sustainability (Shah et al., 2021).

With the wide application of total factor productivity analysis in agricultural development (Liu and Feng, 2019), especially the proposal of green total factor productivity (GTFP), researchers can better measure and study sustainable development of agriculture (Chen et al., 2021). GTFP is also regarded as an ideal indicator for studying sustainable development of agriculture (Chen et al., 2021; Liu et al., 2022). GTFP takes the negative impact of agricultural production on the environment as an undesirable output (Tugcu and Tiwari, 2016; Yu et al., 2022), and incorporates it into the calculation framework. Stochastic Frontier Analysis (SFA) and Data Envelopment Analysis (DEA) are the main methods to measure GTFP (Tang et al., 2017; Shi and Li, 2019; Baležentis et al., 2021). DEA does not rely on the form of production function, and can be adapted to the efficiency calculation of complex systems with multiple input and output variables (Johnes, 2015; Song et al., 2018; He et al., 2021). Considering the diversity of factor inputs and undesirable outputs in agricultural production, DEA method is more suitable for measuring GTFP (Liu and Feng, 2019).

As to the improvement of GTFP, current research mostly discusses it from the perspective of output side and input side. The first is to increase agricultural output and reduce undesirable output at the output end. For example, relevant studies believe that environmental regulation can reduce unexpected output, thereby improving green production efficiency (Xie et al., 2017; Tang et al., 2022). The second is to reduce the use of pollutants at the input side and improve the utilization efficiency of energy chemicals. The innovation of physicochemical technology can improve the utilization efficiency of input factors such as chemical fertilizers and pesticides (Wang et al., 2021a). The promotion of green technologies (or clean technologies) can optimize the allocation of production factors, reduce the use of pollutants such as chemical fertilizers and pesticides, and effectively improve the conversion efficiency of energy chemicals (Midingoyi et al., 2018; Eanes et al., 2019). In sustainable agriculture practice, improvement of agricultural production efficiency through green technologies has drawn much attention (Shah et al., 2021; Rahman et al., 2021). Green technologies are involved in all sectors of grain production, processing, storage and transportation (MARAPRC, 2018). Among them, green technologies in the production link include that in plowing, sowing, fertilization, irrigation, etc. (Midingoyi et al., 2018; He et al., 2021; Mao et al., 2021; Zhao et al., 2021). These green technologies have played a significant role in improving grain production and reducing greenhouse gas emissions and non-point source pollution (Zhuang et al., 2019; Zhu et al., 2021; Chen et al., 2022).

With the greatest population, China has made remarkable progress in the continuous improvement of agricultural production value and grain output (Liu and Feng, 2019), achieving food security for 18% of the world’s population with only 9% of the world’s arable land and 6% of the world’s water resources (Wang et al., 2018). With the increasing pressures of population growth, resource shortage, carbon emissions and environmental destruction, the green production of grain has been an important approach to achieve sustainable food security in China (Rahman et al., 2021; ECOSOC, 2022). However, literatures on the green production of grain are still relatively insufficient, especially in green productivity estimation and its influencing factors. The existing researches showed that China’s green productivity of grain shows a trend of fluctuating growth (Xue and Gu, 2022), which was affected by agricultural labor force, technological innovation, storage policy and other factors (Wang and Yang, 2020; Gao, 2022; Li and Lin, 2022). Nevertheless, China’s agriculture is dominated by extensive management, and agricultural development and food security are achieved at the cost of high energy consumption and serious environmental pollution (Yang et al., 2018). Compared with the mechanized production and industrialized operation in developed countries such as the United States, China’s grain production features small-scale farmers (Guo et al., 2022b), a lower degree of mechanization (Qiu and Luo, 2021), and large gap in relevant technical level and management experience with those countries (Si et al., 2021). This leads to the negative impact of China’s agricultural mechanization on energy-environment efficiency (Jiang et al., 2020). There is a long-term correspondence between the improvement of agricultural mechanization and increase of energy consumption and carbon emissions (Fabiani et al., 2020), the application of green technologies in agricultural mechanized production is of great significance for improving GTFP (Li et al., 2020; He et al., 2021).

Actually, the impacts of technology application on GTFP are nonlinear (Luan et al., 2019; He et al., 2021). Due to the lag effect of technology application (Mao et al., 2021), green technologies can only play a role in increasing production, improving environment and reducing pollution after a period of application (Huisingh et al., 2015; Chen et al., 2017; Gao et al., 2018).When machinery and energy are overused, the continuous application of green technology will also lead to low productivity and increased carbon emissions (Silva-Olaya et al., 2013; Zhao et al., 2018; Min et al., 2021), that is, the impacts of green technology application on GTFP has a threshold effect. As an important part of agricultural modernization (Houssou et al., 2013), mechanization has made great contributions to China’s food security (Min et al., 2021; Yang et al., 2022a). The application of green technologies in agricultural mechanization also has threshold restrictions. Appropriate application conditions and adoption rates will promote green production (Zhang et al., 2020; He et al., 2021), while inappropriate conditions and excessive application will also have negative impacts on the environment (Min et al., 2021; He et al., 2021). Therefore, studying the threshold impacts of green technologies on grain green productivity in agricultural mechanization has great significance (He et al., 2021), which is very scarce and necessary.

The main contributions of this paper are as follows: according to the difference of ecological effects, the application of green technology was divided into the initial development stage, the ecological efficiency stage and the overuse stage, so as to build a mechanism framework of the nonlinear effects of green technology application on the green total factor productivity of grain (GTFPG); it took carbon emissions and non-point source pollution as undesirable output, and used the Super Epsilon Based Measure (Super-EBM) model and the Global Malmquist Lunberger (GML) productivity index to calculate the GTFPG, the green technology efficiency change of grain (GECG) and the green technical progress change of grain (GTCG); the correlations between GTFPG and green technologies were analyzed with pattern evolution, kernel density curve and box plot; by matching the ecological effects of green technologies in different application stages with their threshold results, we could better comprehend the nonlinear effects of green technologies on GTFPG; besides, the paper investigated the different influence paths of various green technologies on GTFPG in the major and non-major grain producing areas, and revealed that regional technology preference, resource endowment and technology compatibility diversified the influence paths of green technologies. The paper highlights the threshold effect of green technology application on GTFPG. Therefore, the development of green technologies should take into full account the productivity, resource endowment of different regions, and applicability of green technologies. Adoption rate of green technologies should be reasonably controlled. It is hoped that this paper will provide theoretical basis and practical experience for sustainable development of grain in China.

## 2 Materials and methods

### 2.1 Green total factor productivity and its decomposition

GTFP has been widely used as an ideal index to measure agricultural green development (Chen et al., 2021; Chen et al., 2021; Liu et al., 2022). To calculate GTFP, first green production efficiency is obtained through DEA, and then GTFP and its decomposition (GECG and GTCG) are obtained through GML productivity index.

#### 2.1.1 Green productivity

The production frontier function of DEA model may be parallel to the coordinate axis, resulting in disparities between the DMU falling on these parallel functions and the strong effective target value, including the Proportionate Movement part and the Slack Movement part. However, the radial DEA model can only solve the Proportionate Movement part, which leads to the deviation of the efficiency measurement value. Therefore, the non-radial DEA model can fully consider the Slack Movement part, realize the compatibility of the Proportionate Movement part and the Slack Movement part, and ensure the original information of the efficiency frontier’s projection values (Cheng, 2014). Therefore, the paper chooses the Epsilon Based Measure (EBM) model constructed by Tone and Tsutsui (2010) to measure green productivity. Meanwhile, in order to distinguish the differences between decision-making units (DMUs) with the same efficiency of 1, the paper finally follows the research methods of Wu et al. (2020) and Zhao et al. (2022), and uses the Super-EBM model to calculate the green productivity of grain. The Super-EBM model can be expressed as:


(1) $$\tilde{E}=\min\left[\frac{\theta -\epsilon_{x}\sum_{i=1}^{m}\frac{w_{i}^{-}s_{i}^{-}}{x_{ik}}}{\phi +\epsilon_{y}\sum_{r=1}^{q}\frac{w_{r}^{g+}s_{r}^{g+}}{y_{rk}}+\epsilon_{v}\sum_{t=1}^{p}\frac{w_{t}^{b-}s_{t}^{b-}}{v_{tk}}}\right]$$

(2) $$\{\begin{array}{l} \sum_{j=1,j\neq k}^{n}x_{ij}\lambda_{j}-s_{i}^{-}\leq \theta \cdot x_{ik}\begin{array}{cc} , & i=1,\ldots ,m \end{array}\\ \sum_{j=1,j\neq k}^{n}y_{rj}\lambda_{j}-s_{r}^{g+}\geq \phi \cdot y_{rk}\begin{array}{cc} , & r=1,\ldots ,q \end{array}\\ \sum_{j=1,j\neq k}^{n}v_{tj}\lambda_{j}-s_{t}^{b-}\leq v_{tk}\begin{array}{cc} , & t=1,\ldots ,p \end{array}\\ \begin{array}{cccc} \lambda \geq 0, & s^{-}\geq 0, & s^{g+}\geq 0, & S^{b-}\geq 0 \end{array} \end{array}$$

where $\tilde{E}$ represents the value of green productivity of grain; *x_ij_* is the input variable matrix, with specific indicators including planting area, fertilizer, pesticide, agricultural film, diesel oil, seed, electricity for irrigation, labor and machinery (Liu and Feng, 2019; He et al., 2021; Li and Lin, 2022); *y_rj_* represents the desirable output, which is expressed in grain production; *v_tj_* represents the undesirable output, including carbon emissions and non-point source pollutions (the measurement of carbon emissions follows the methods of Liu et al., 2013 and Chen et al., 2021; and the measurement of non-point source pollutions follows the methods of Chen et al., 2006 and Zou et al., 2020); $s_{i}^{-}$ , $s_{r}^{g+}$ and $s_{t}^{b-}$ are slacks of inputs, slacks of desirable outputs and slacks of undesirable outputs respectively; $w_{i}^{-}$ , $w_{r}^{g+}$ and $w_{t}^{b-}$ represent the relative importance of various input indicators, desirable outputs and undesirable outputs, with $\sum_{i=1}^{m}w_{i}^{-}=1(w_{i}^{-}\geq 0)$ , $\sum_{i=1}^{m}w_{r}^{g+}=1(w_{r}^{g+}\geq 0)$ and $\sum_{i=1}^{m}w_{t}^{b-}=1(w_{t}^{b-}\geq 0)$ ; *θ* represents the efficiency value under input orientation; *φ* represents the efficiency value under output orientation; *ε* is the importance of the non-radial part, *ε*∈ [0, 1].

#### 2.1.2 Global malmquist-luenberger productivity index

In order to better reflect the change state of productivity, this paper measures the green total factor productivity of grain (GTFPG) with the help of the Global Malmquist Lunberger (GML) index proposed by Oh (2010) based on the calculation of green productivity by the Super-EBM model, and decomposes it into the green technology efficiency change of grain (GECG) index and the green technical progress change of grain (GTCG) index, then GTFPG = GECG×GTCG. GTFPG, GECG and GTCG can be expressed as:


(3) $$GECG_{i,t+1}=\frac{1+E_{c}^{it}(x^{it},y^{it},b^{it})}{1+E_{C}^{i,t+1}(x^{i,t+1},y^{i,t+1},b^{i,t+1})}$$

(4) $$GTCG_{i,t+1}=\frac{1+E_{C}^{i,t+1}(x^{i,t+1},y^{i,t+1},b^{i,t+1})}{1+E_{G}^{it}(x^{i,t+1},y^{i,t+1},b^{i,t+1})}\times \frac{1+E_{G}^{it}(x^{it},y^{it},b^{it})}{1+E_{C}^{it}(x^{it},y^{it},b^{it})}$$

(5) $$GTFPG_{i,t+1}=\frac{1+E_{G}^{it}(x^{it},y^{it},b^{it})}{1+E_{G}^{it}(x^{i,t+1},y^{i,t+1},b^{i,t+1})}=GEC_{i,t+1}\times GTC_{i,t+1}$$

where the values of GTFPG, GECG and GTCG are greater than 0; when GTFPG > 1, means the GTFPG increases; on the contrary, means the GTFPG decreases. The values of GECG and GTCG have the same meaning.

### 2.2 Green technologies and the mechanism of their impacts

#### 2.2.1 Selection of green technologies

Based on the research of He et al. (2021) and Zhai et al. (2021), this study selects six green technologies from plowing, sowing, fertilization and irrigation in agricultural mechanized production. They are the mechanical deep-plowing and subsoiling (MDPS) in plowing stage, the precision and small quantity sowing (PSQS) and mechanized no-tillage sowing (MNTS) in sowing stage, the mechanized straw returning(MSRE) and mechanical fertilizer deep distributing (MFDD) in fertilization stage, and the water-saving irrigation (WSIR) in irrigation stage. Application of green technologies requires certain conditions. Suitable conditions and appropriate adoption ratio will promote their ecological effects (Gao et al., 2018; Zhang et al., 2020; He et al., 2021), while mismatched conditions and excessive application will have a negative impact on the environment (Zhao et al., 2018; Wang et al., 2020a; Min et al., 2021; He et al., 2021). The application conditions (Yang et al., 2019; CPGPRC, 2007; Duan et al., 2022a; Yin et al., 2016; Tian et al., 2019; Zhao et al., 2020; Wang, 2021; Chen et al., 2022; Zhuang et al., 2019), ecological effects (CPGPRC, 2007; Shao et al., 2016; Wang et al., 2015; Wang et al., 2020a; Chen et al., 2020; Karayel, 2009; Li et al.,2015; Chaudhary et al., 2021; Keshavarz Afshar and Dekamin, 2022; Zhang et al., 2022; Sun et al., 2018; Hu et al., 2022; Zhu et al., 2021; Zhong et al., 2021; Wu et al., 2021; Gaihre et al., 2015; Miah et al., 2016; Man et al., 2014; Wang et al., 2020b; Zhuang et al., 2019) and negative impacts (Baumhardt et al., 2008; Ding et al., 2018; CPGPRC, 2007; Chen et al., 2022; Rahim et al., 2021; Peixoto et al., 2020; Sun et al., 2022; Pisante et al., 2014; Wang et al., 2021b; Wang et al., 2021a; Li et al., 2018; Hu et al., 2022; Yang et al., 2020; Zhao et al., 2021; Xia et al., 2022; Zhuang et al., 2019; Wang et al., 2022) of each green technology are shown in **Table S1**.

#### 2.2.2 The influence mechanism of green technology application on GFTPG

**Figure 1:**
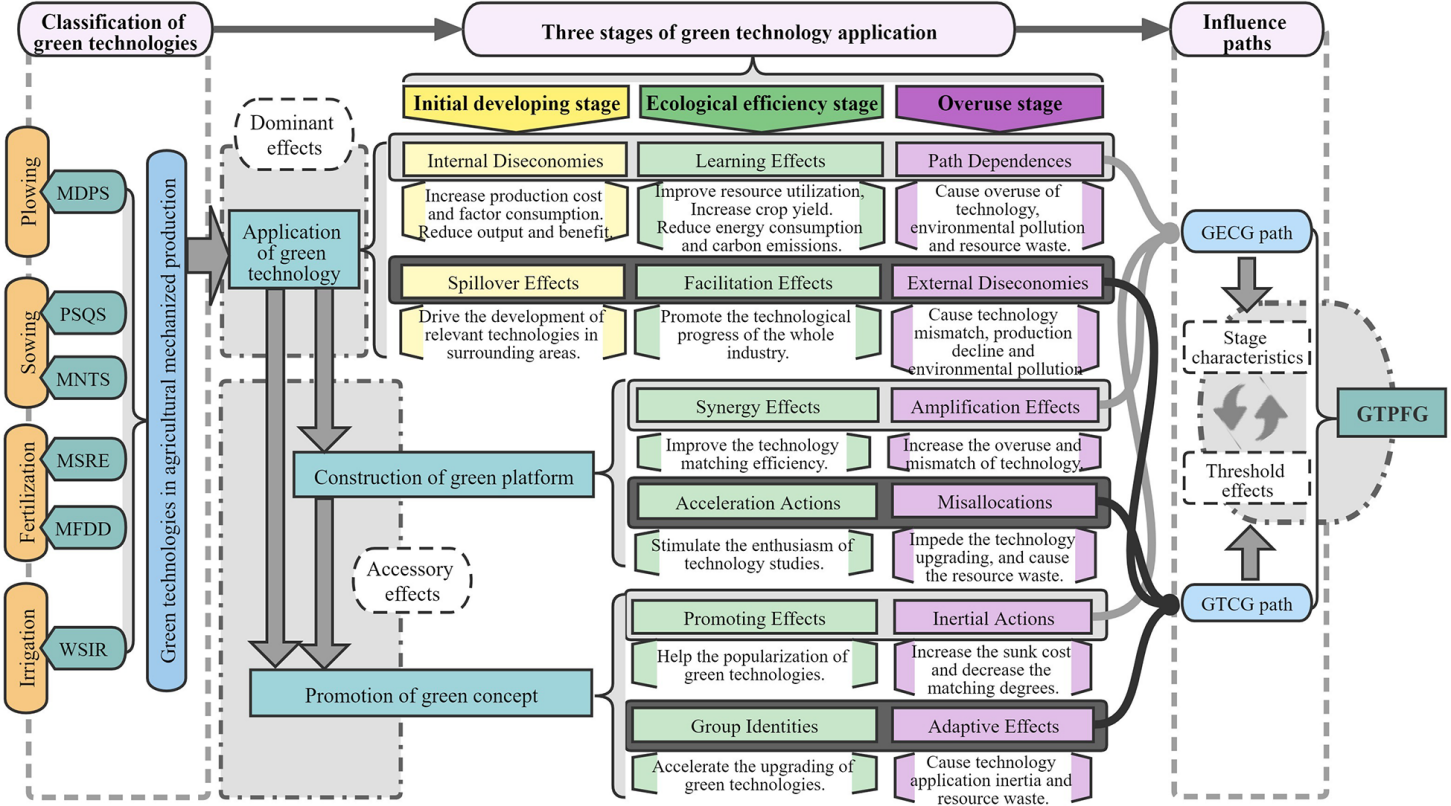
The influence mechanism of green technology application on GFTPG.

Efficient and environment-friendly, green technologies have a long-term industrial ripple effect on the agricultural sector and have become an important part of the green development platform, affecting people’s perception of sustainable development and research on GFTPG (Wang et al., 2021a; Zhu et al., 2021; Chen et al., 2022). This paper decomposes GFTPG into the green technology efficiency change of grain (GECG) and the green technical progress change of grain (GTCG), and discusses the impacts of technology application, as well as platform construction and perception promotion driven by it, on GECG and GTCG respectively. Green technology application is divided into different stages according to its ecological effects, to better understand the influence mechanism of green technologies on GFTPG. The influence mechanism of green technology application on GFTPG is shown in **Figure 1**.

The impacts of green technology application on GECG. First, in the initial stage of green technology application, the Diseconomies of Scale may lead to the decline of GECG (Zhong et al., 2022). The Diseconomies of Scale is reflected in the increase of average cost and decrease of yield and income, due to the allocation of new equipment, low technical level and lack of management experience (Zhang et al., 2016; Si et al., 2021; Zhong et al., 2022), that is, the Internal Diseconomies of technology application. Secondly, with increasing application rate, green platform is gradually formed (Reza-Gharehbagh et al., 2022), which helps to connect technology developers and users and provide supporting social services, thereby accelerating the application of green technology and reducing cost (Totin et al., 2020), namely the Learning Effects of technology application and the Synergy Effects of green platform. Additionally, the green platform will bring inertia to the application of green technology (Inertial Actions). Combined with the sunk cost and application threshold of new technology (Zhang et al., 2016; Mañez and Love, 2020), it will increase the Path Dependences of green technology application, and aggravate the overuse and mismatch of technologies (Amplification Effects), thereby reducing GECG. Finally, the large-scale application of green technologies has accelerated promotion of green concepts. More subjects have participated in green production, which promotes popularization of green technologies and improvement of regional GECG (Promoting Effects). However, attention should also be paid to the contradiction between standardized machinery and diversified demand in green production, as well as the mismatch between rising technology adoption rate and the level of social services (Zhuang et al., 2019; Wang et al., 2021b; Zhang et al., 2021), all of which will hinder the improvement of GECG.

The impacts of green technology application on GTCG. First, the development and application of green technologies has Spillover Effects (Pan et al., 2021), which is reflected in the fact that the increase of green technology adoption rate in a region will promote the development of related technologies in its surrounding areas, and can trigger the Facilitation Effects of technology application. However, the Spillover Effects may also lead to overuse of the technology throughout the planting industry. Pursue the growth of green technology adoption rate while ignoring the application threshold and applicable conditions ultimately leads to the grain output decrease and environmental pollution, that is, the External Diseconomies of technology application (Zhong et al., 2022). Secondly, the green platform built for the application of green technology can arouse the enthusiasm of participants (especially the proportion of ordinary farmers), strengthen rural collective action, stimulate the initiative of enterprises in green technology research and development, promote the application and integration of green technology in various production links (Wang et al., 2022), and thus accelerate the development and promotion of new technologies (Acceleration Actions). However, excessive emphasis on the role of green platforms will also lead to resource Misallocations. Finally, the high adoption rate of green technologyies reflects the Group Identities with green development concept, which can effectively promote the upgrading and progress of the overall agricultural green technology, and promote the improvement of GTCG. However, when the concept of green development does not match the actual production, it cannot effectively improve GTCG (Adaptive Effects). For example, when mechanical equipment, operation level, natural conditions and social services cannot support green technology to exert its ecological effect, excessive pursuit of high adoption rate will lead to increase of cost, decrease of benefit and waste of resources (He et al., 2021; Min et al., 2021), which will also lead to External Diseconomies of technology application.

The impacts of green technology adoption on GTFPG. The analysis above shows that the impacts of adoption rate of green technologies on GECG and GTCG has threshold effects, which will also be reflected in the impacts on GTFPG. The threshold effects can be understood as its different impacts on GTFPG at different stages: the initial development stage, ecological efficiency stage and overuse stage. Specifically, in the initial developing stage, the application of green technologies is in the experimental and demonstration period. Construction of green platforms and promotion of green concepts just get started. Supporting machinery, technology, management and social services are at a low level. The resulting Diseconomies of Scale (Internal and External Diseconomies) hinder the improvement of GTFPG. In the ecological efficiency stage, technology, machinery configuration and management are improved. Green platforms are enhanced, green concept is widely recognized, and rural collective action is further strengthened. The marginal cost of production is reduced, and resource allocation is more reasonable. Green technology is upgraded and promoted faster, and starts to exhibit its ecological effect, thus effectively improving GTFPG. During the overuse stage of green technologies, due to the high level of green technology application, platform construction and concept identity in the area with high green technology adoption rate, and the sunk cost of technology application decision, Path Dependences may occur, which hinders the upgrading and improvement of green technologies. Meanwhile, ignoring the improvement of quality and efficiency of traditional agricultural technology, as well as the resource allocation in areas where green technology is not applicable, may also cause resource Misallocations, and lead to Diseconomies of Scale (Internal and External Diseconomies), which hinders the improvement of GTFPG. Due to the differences in application conditions, applicability and negative effects of various green technologies, theirs impacts on GTFPG have different threshold effects.

### 2.3 Model construction and data sources

#### 2.3.1 Threshold model

Considering that the effects of green technology application have hysteresis (Chen et al., 2017; Gao et al., 2018; Mao et al., 2021), and require appropriate application conditions and proportions (Zhang et al., 2020; Min et al., 2021), otherwise it will have negative impacts on the environment (Wang et al., 2020a; He et al., 2021), thus, there are threshold effects of green technology on GTFPG. This paper refers to the threshold model proposed by Hansen (1999) to explore the nonlinear effects of green technology application on GTFPG. The single-threshold model can be expressed as:


(6) $$y_{it}=\alpha_{i}+\beta_{1}X_{it}I(thre_{it}\leq \gamma )+\beta_{2}X_{it}I(thre_{it}>\gamma )+\epsilon_{it}$$

where *X_it_* represents the set of explanatory variables; *β*_1_ and *β*_2_ are coefficient estimates; *thre_it_* represents the threshold variable (which can be a part of *X_it_*), and *γ* is the threshold value; *I*(*) is the indicating function, when the inequality in brackets is ture, *I*(*)=1, otherwise, *I*(*)=0.

The double-threshold model can be expressed as:


(7) $$y_{it}=\alpha_{i}^{\prime }+\beta_{1}^{\prime }X_{it}I(thre_{it}^{\prime }\leq \gamma_{1})+\beta_{2}^{\prime }X_{it}I(\gamma_{1}<thre_{it}^{\prime }\leq \gamma_{2})+\beta_{3}^{\prime }X_{it}I(thre_{it}^{\prime }>\gamma_{2})+\epsilon_{it}$$

where $\beta_{1}^{\prime }$ , $\beta_{2}^{\prime }$ and $\beta_{3}^{\prime }$ are coefficient estimates; $thre_{it}^{\prime }$ represents the threshold variable, *γ*_1_ and *γ*_2_ represent two threshold values.

#### 2.3.2 Control variables selection

Considering that the GTFPG is affected by various factors, in order to remove the interference of other factors on green technology, this paper uses the research approaches of Xu et al. (2020); He et al. (2021); Yang et al. (2022b), and Li and Lin (2022) selects control variables from production condition, production decision, agglomeration capacity, financial support, economic development and natural disaster. Production condition increases the marginal desirable output or reduce the undesirable output by matching with the productivity level (Jiang et al., 2020; Li and Lin, 2022), and agricultural mechanization level and irrigation level are selected as proxy variables; production decision affects productivity by changing the proportion of production elements and production scales (Jiang et al., 2020; Liu et al., 2020; Li et al., 2020), and planting structure and rural income level are selected as proxy variables; agglomeration capacity improves resource utilization efficiency through knowledge spillover and energy structure optimization (Li and Lin, 2022; Yang et al., 2022b), and grain production agglomeration is selected as the proxy variable; financial expenditure affects productivity by improving production input, management level and service quality (Chen et al., 2021; He et al., 2021), and agricultural fiscal level and agricultural investment level are selected as proxy variables; economic development improves green productivity by influencing the adoption of green technologies and environmental awareness (Xu et al., 2020; Liu et al., 2022), and urbanization level and trade dependence level are selected as proxy variables; natural disasters have directly led to the decline of grain output and the increase of energy and chemical products input (Chen et al., 2021; He et al., 2021; Liu et al., 2022), and disaster incidence level, temperature fluctuation level and precipitation fluctuation level are selected as proxy variables. The specific calculation method of each control variable is shown in **Table S2**.

#### 2.3.3 Data sources

**Figure 2:**
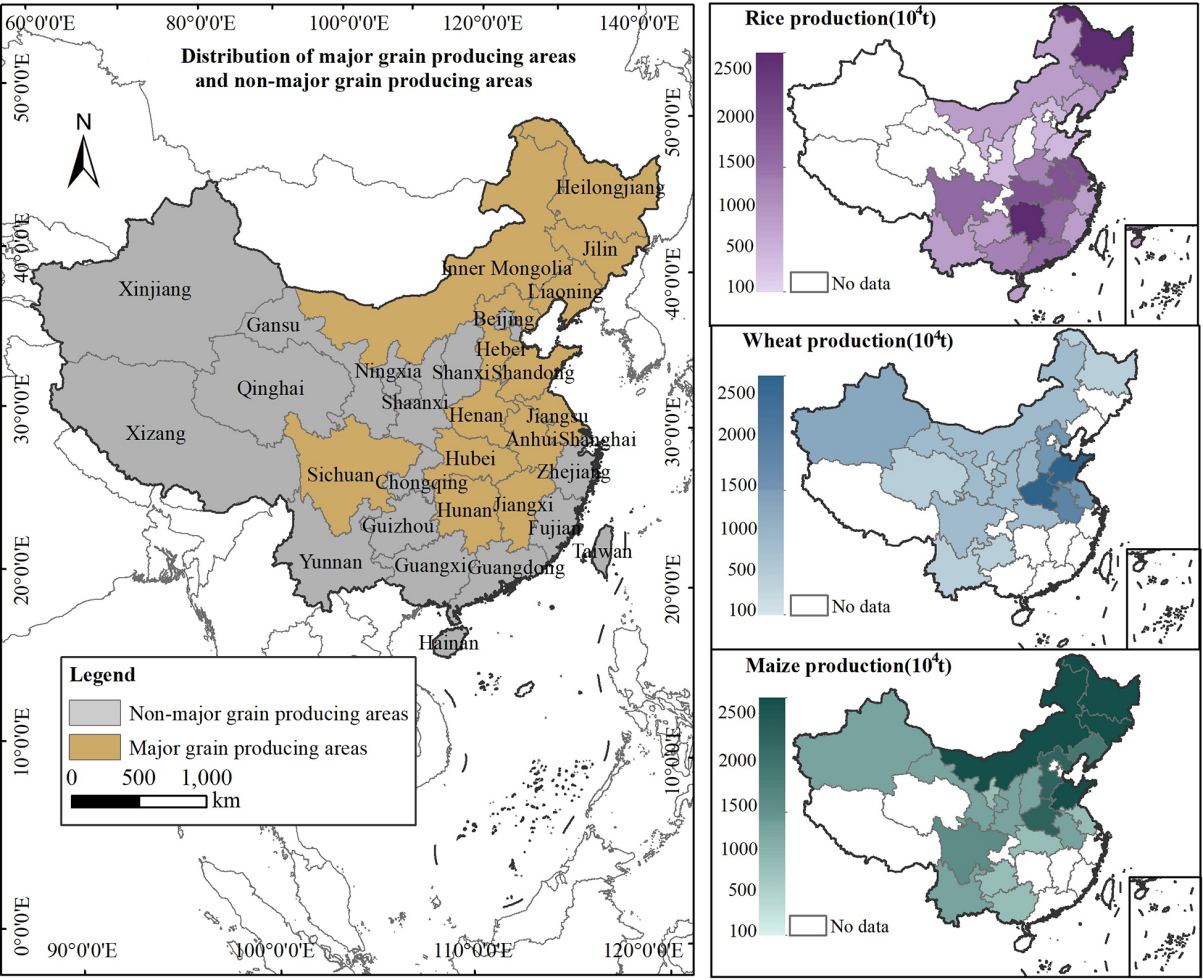
Distribution of the research areas.

The paper selects rice, wheat and maize as the representative varieties of grain. In 2020, the total output of rice, wheat and maize was 606.7786 million tons, accounting for 90.633% of the total grain output, so it can substitute for grain crops for research. Limited by the availability of green technology data, the sample period selected in this paper is from 2000 to 2020. Tianjin, Hebei, Shanxi, Inner Mongolia, Liaoning, Jilin, Heilongjiang, Jiangsu, Zhejiang, Anhui, Fujian, Jiangxi, Shandong, Henan, Hubei, Hunan, Guangdong, Guangxi, Hainan, Sichuan, Guizhou, Yunnan, Shaanxi, Gansu, Qinghai, Ningxia and Xinjiang are selected as the research areas. In 2020, the staple food (rice, wheat and maize) output of these 27 provinces was 582.442 million tons, accounting for 95.989% of China’s total staple grain output, so the samples are highly representative. Additionally, Chinese government set up major grain producing areas, funds, technology, talent flew to these areas and promoted the annual growth of grain production (Yang et al., 2021; Li and Lin, 2022). Therefore, it is necessary to examine the changes of green total factor productivity of grain in the major grain producing areas and non-major grain producing areas respectively (He et al., 2021). The major grain producing areas were divided according to the definition of SCPRC (2017). The distribution of these areas is shown in **Figure 2**.

The data of green technologies comes from the China Agricultural Machinery Industry Yearbook; the data of staple grain production and planting area are from the National Bureau of Statistics of China; the input data in the grain production process are from the Ministry of Agriculture and Rural Affairs of China, the China Rural Statistical Yearbooks and the provincial Statistical Yearbooks; the original data of the control variables are from the National Bureau of Statistics of China and the provincial Statistical Yearbooks, and are calculated according to **Table S3**. In order to eliminate the inflation impacts, the data measured in monetary units in this paper are reduced by the consumer price index (CPI) based on 2000 to obtain the real values. The descriptive statistics of each variable are shown in **Table S3**.

## 3 Results and analysis

### 3.1 Measurement of GTFPG, GECG and GTCG

#### 3.1.1 Spatial-temporal pattern analysis

**Figure 3:**
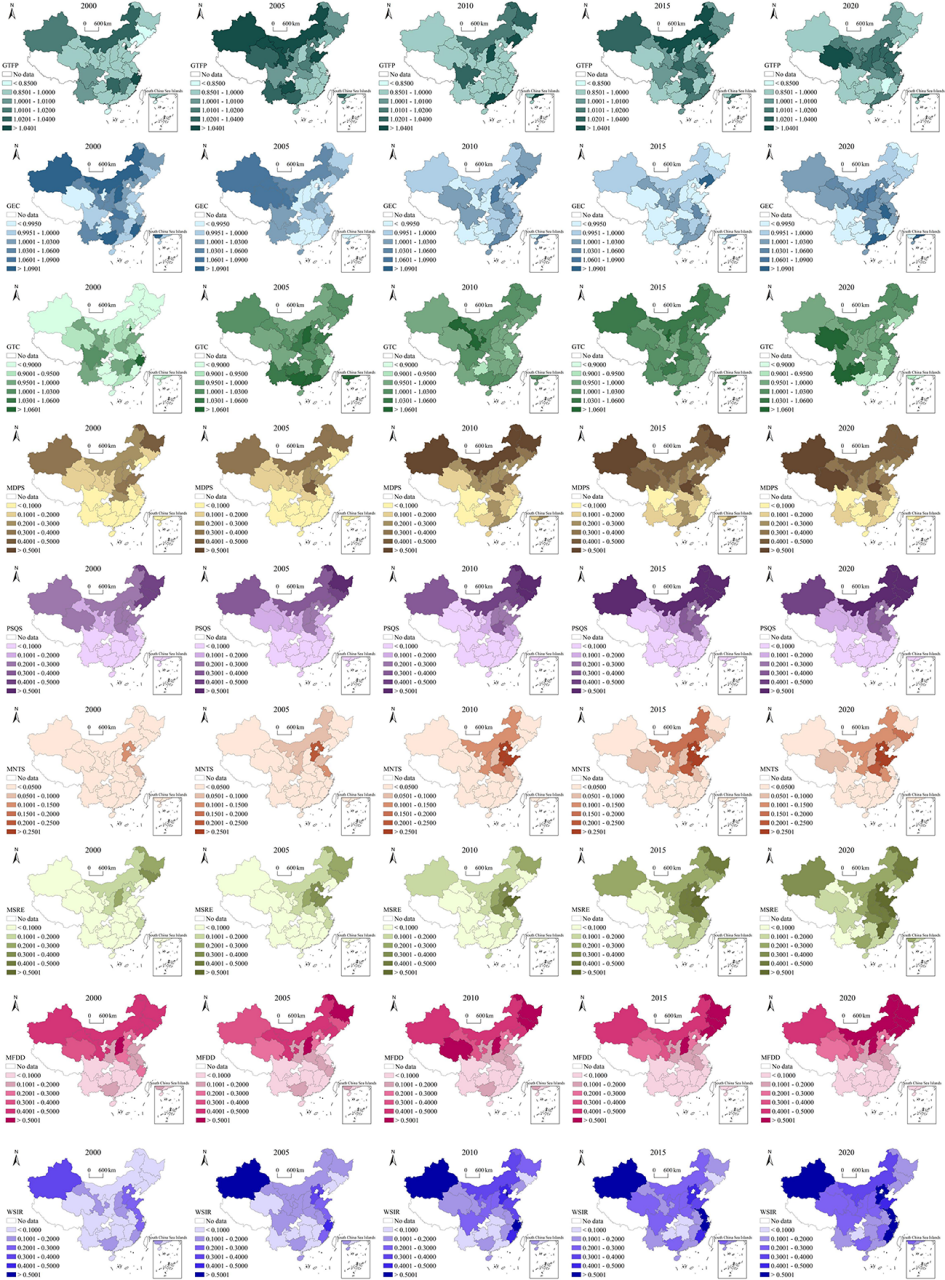
Spatial-temporal pattern of GTFPG, GECG, GTCG and green technologies from 2000 to 2020.

China’s GTFPG shows an ‘N’ shaped trend from 2000 to 2020 (**Figure 3**). GTFPG was on the rise from 2000 to 2005, and that of grain planting areas in northern China was significantly higher than that in southern China. From 2005 to 2010, overall GTFPG decreased significantly, and that in Inner Mongolia-Northeast China was higher. From 2010 to 2015, overall GTFPG gradually recovered, and that in Inner Mongolia-Northeast China grain producing areas was still high, while that in the middle and lower reaches of the Yangtze River areas (Anhui, Hubei, Hunan) increased significantly. From 2015 to 2020, areas with higher GTFPG gathered along the Yellow River (Qinghai, Shanxi, Shaanxi, Gansu, Ningxia) and Huang-Huai-Hai area (Hebei, Henan, Shandong). China’s GECG showed a ‘U’ shaped trend from 2000 to 2020 (**Figure 3**). The regions with higher GECG gathered to the provinces along the Yellow River, the lower reaches of the Yangtze River, and the Huang-Huai-Hai grain production areas. China’s GTCG showed an ‘M’ shaped trend from 2000 to 2020. The regions with higher GTCG gathered in southwestern China (Yunnan, Guizhou) and central and western China (Qinghai, Sichuan).

The application of various green technologies has obvious spatial agglomeration (**Figure 3**). From 2000 to 2020, MDPS had a higher adoption rate in the northern areas than in the south, and formed the distribution pattern centered around Xinjiang, Northeast producing areas (Heilongjiang, Jilin) and the areas along the Yellow River. PSQS had a higher adoption rate in the northern producing areas than in the southern areas, and formed the distribution pattern centered on the Inner Mongolia-Northeast producing areas. Besides, its adoption rate in Xinjiang and Huang-Huai-Hai area was also relatively high. MNTS gradually formed the distribution pattern centered on the Huang-Huai-Hai area. The adoption rate of MNTS in the arid and semi-arid regions of the north was significantly higher than that in the south. MSRE formed the distribution pattern centered on the Huang-Huai-Hai area and the lower reaches of the Yangtze River. MFDD had significantly higher adoption rate in the north than in the south, and formed the distribution pattern centered on the Inner Mongolia-Northeast region. The distribution of WSIR is centered on the eastern coastal provinces, and it had high adoption rate in Xinjiang.

Comparison of the spatial distribution of GTFPG and green technologies shows that, in different periods, there is correlation between GTFPG and green technologies in terms of spatial evolution, as well as obvious regional differences. For example, the concentration of high GTFPG in some northern producing areas from 2000 to 2005 may be correlated with changes in the adoption rates of MDPS, MNTS, and MSRE in these provinces. However, the change of GTFPG is also affected by financial support, agricultural investment, natural disasters, etc. Therefore, there is no simple correspondence between GTFPG and the change of green technology adoption rates. Investigation into the impacts of green technologies on GTFPG and the influence paths requires fitting analysis through multiple regressions.

#### 3.1.2 Regional differences analysis

**Figure 4:**
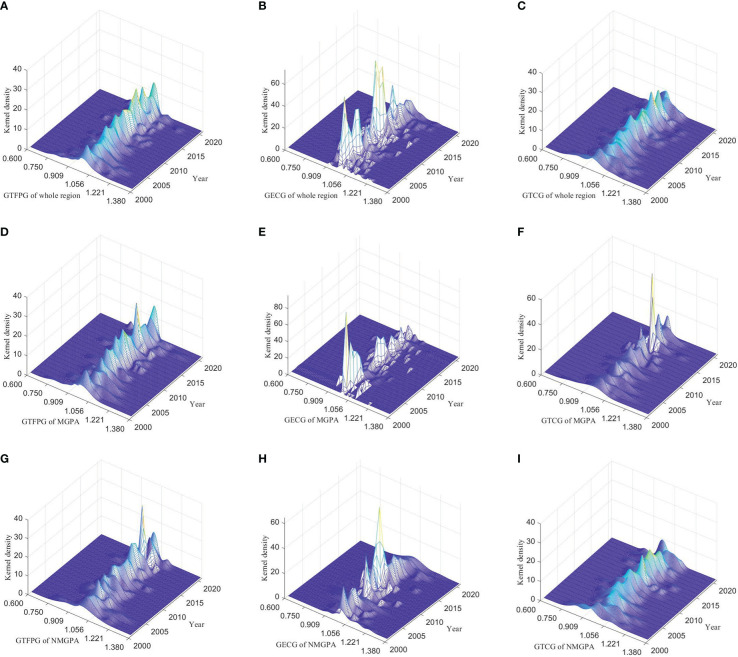
Three-dimensional kernel density curves of GTFPG, GECG and GTCG. MGPA: major grain producing areas; NMGPA: non-major grain producing areas. **(A–C)** are kernel density curves of GTFPG, GECG and GTCG of the whole region respectively. **(D–F)** are kernel density curves of GTFPG, GECG and GTCG of the major grain producing areas respectively. **(G–I)** are kernel density curves of GTFPG, GECG and GTCG of the non-major grain producing areas respectively.

This paper further analyzes the dynamic difference of sustainable grain production in China by means of three-dimensional kernel density function. The kernel density curves of GTFPG, GECG and GTCG in the whole region are shown in **Figures 4A– C**. The integral area of GTFPG kernel density curve in the whole region changed from ‘low-wide’ to ‘high-narrow’, which means that provincial difference of GTFPG shrank. The peak of the kernel density curve of GECG is significantly higher than that of GTFPG, indicating small regional differences of GECG. The peak of the kernel density curve of GTCG in the whole region increased first and then decreased, which reflects that the regional difference of GTCG decreases first and then increases. Similarly, it can be concluded that the regional difference of GTFPG in the major grain producing areas decreased (**Figure 4D**); the regional difference of GECG in the main grain producing areas has increased, but the overall level was still low (**Figure 4E**). The increase of GECG agglomeration in the major grain producing areas leads to the decrease of regional differences of GTFPG (**Figure 4F**). The regional difference of GTFPG in the non-major grain producing areas is greater than that in the whole region and in the major grain producing areas (**Figure 4G**). The overall regional difference of GECG in the non-major grain producing areas is small (**Figure 4H**). The regional difference of GTCG in the non-major grain producing areas is large (**Figure 4I**). In summary, the difference of GTFPG among provinces in China gradually decreases, which is mainly attributed to the regional differences of GTCG, while the regional differences of GECG remain small.

**Figure 5:**
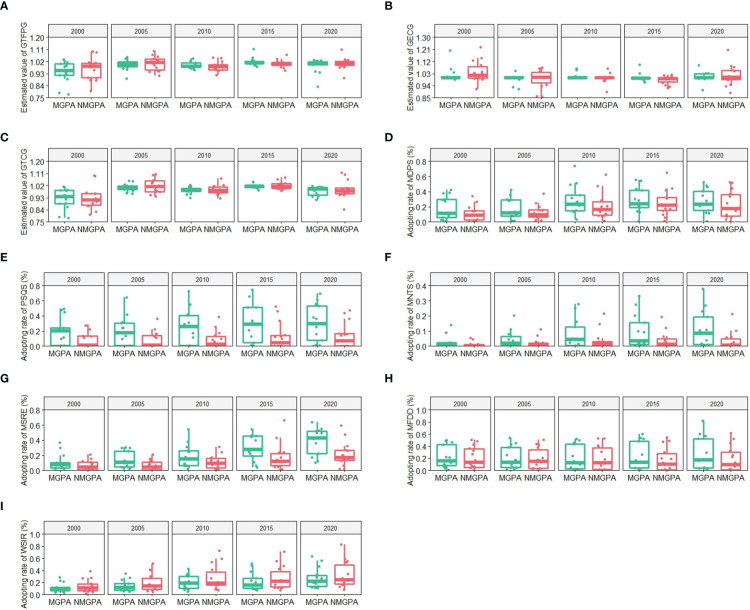
Regional difference of GTFPG, GECG, GTCG and green technologies from 2000 to 2020. MGPA: major grain producing areas; NMGPA: non-major grain producing areas. **(A–C)** are estimated values of GTFPG, GECG and GTCG in the major and non-major grain producing areas respectively. **(D–I)** are adoption rates of MDPS, PSQS, MNTS, MSRE, MFDD and WSIR in the major and non-major grain producing areas respectively.

Based on the analysis of the difference of the whole sample through kernel density curve, this paper uses box plot to analyze the differences in GTFPG, GECG, GTCG and green technology adoption rate between major grain producing areas and the non-major producing areas, as shown in **Figure 5**. Firstly, differences in the change of GTFPG from 2000 to 2020 show that sample dispersion of GTFPG in both major and non-major grain producing areas was significantly reduced (**Figure 5A**). In 2000, the median of GTFPG in the major grain producing areas (0.954) was significantly lower than that in the non-major grain producing areas (0.985). In 2020, the median of GTFPG in the major grain producing areas (1.005) increased slightly and was roughly equal to that in the non-major grain producing areas (1.002). The median of GECG in the major grain producing areas and non-grain producing areas shows a fluctuating upward trend (**Figure 5B**). The median of GTCG increased first and then decreased (**Figure 5C**).

Secondly, differences in green technology adoption rate from 2000 to 2020 show that major grain producing areas and non-major grain producing areas have obvious different preference for green technologies. In summary, the adoption rate of all green technologies in the major grain producing areas has increased. Compared with non-major grain producing areas, the adoption rates of MDPS, PSQS, MNTS and MSRE in the major grain producing areas are higher. The adoption rates of MDPS and WSIR in the non-major grain producing areas have increased more significantly, and that of MSRE has increased faster than that in the major grain producing areas (**Figures 5D–I**). In order to study quantitatively the impacts, influence paths and regional differences of green technologies, this paper further analyzes the threshold results of GTFPG, GECG and GTCG.

### 3.2 Threshold analysis of GTFPG

Before we use the threshold model for empirical analysis, it is necessary to test the threshold effect of the constructed model, and the test results are shown in **Tables S4–S6**. All green technologies (MDPS, PSQS, MNTS, MSRE, MFDD, WSIR) show threshold effects on GTFPG, GECG and GTCG, reflecting that application of green technologies has a non-linear effect on GTFPG as well as its decomposition during agricultural mechanization.

#### 3.2.1 Threshold analysis of GTFPG in the whole region

The threshold model results of GTFPG in the whole region are shown in **Table 1**. The results show that all green technologies have double threshold effects on GTFPG. MFDD and WSIR have only positive impact on it. MDPS, PSQS and MSRE have both positive and negative impact, and MNTS has only negative impact. MDPS has the greatest positive impact on GTFPG (1.568). MFDD and WSIR have great positive impact on GTFPG, while NTS and MSRE have great negative impact (only the coefficients that passed the significance test were compared).

**Table 1:** The threshold model results of GTFPG in the whole region.

	Model 1	Model 2	Model 3	Model 4	Model 5	Model 6
MDPS≤γ_1_	-0.436 (0.905)					
γ_1_<MDPS≤γ_2_	1.568 (0.728)**					
MDPS>γ_2_	0.011 (0.024)					
PSQS≤γ_1_		-0.564 (0.607)				
γ_1_<PSQS≤γ_2_		0.082 (0.038)**				
PSQS>γ_2_		0.032 (0.024)				
MNTS≤γ_1_			-0.095 (0.358)			
γ_1_<MNTS≤γ_2_			-1.907 (0.536)***			
MNTS>γ_2_			-0.052 (0.050)			
MSRE≤γ_1_				-2.605 (1.225)**		
γ_1_<MSRE≤γ_2_				0.291 (0.123)**		
MSRE>γ_2_				-0.025 (0.027)		
MFDD≤γ_1_					0.034 (0.057)	
γ_1_<MFDD≤γ_2_					0.164 (0.051)***	
MFDD>γ_2_					0.041 (0.021)*	
WSIR≤γ_1_						0.046 (0.071)
γ_1_<WSIR≤γ_2_						0.147 (0.061)**
WSIR>γ_2_						0.014 (0.027)
UR	0.021 (0.010)**	0.043 (0.019)**	0.028 (0.016)*	0.038 (0.031)	0.022 (0.012)*	0.026 (0.049)
AM	0.219 (0.031)***	0.054 (0.026)**	0.078 (0.014)***	0.019 (0.001)**	0.039 (0.014)***	0.071 (0.019)***
IR	0.088 (0.027)***	0.020 (0.017)	0.042 (0.020)**	0.038 (0.015)**	0.021 (0.015)	0.029 (0.020)
PS	0.031 (0.040)	0.069 (0.040)*	0.049 (0.026)*	-0.020 (0.033)	-0.025 (0.042)	-0.024 (0.040)
AF	-0.044 (0.016)***	-0.063 (0.045)	-0.037 (0.018)**	0.060 (0.075)	-0.013 (0.007)*	0.012 (0.015)
AI	0.054 (0.029)*	0.037 (0.132)	0.116 (0.125)	0.132 (0.127)	0.059 (0.129)	0.132 (0.075)*
RI	0.034 (0.060)	-0.018 (0.069)	0.025 (0.058)	-0.112 (0.059)*	-0.015 (0.062)	0.031 (0.058)
PA	-0.021 (0.012)*	-0.026 (0.012)**	-0.019 (0.017)	-0.009 (0.012)	-0.019 (0.011)*	-0.014 (0.013)
TD	-0.013 (0.013)	-0.013 (0.013)	-0.014 (0.013)	-0.016 (0.009)*	-0.015 (0.013)	-0.011 (0.013)
DI	-0.073 (0.018)***	-0.076 (0.018)***	-0.068 (0.018)***	-0.071 (0.018)***	-0.078 (0.018)***	-0.071 (0.018)
TF	0.016 (0.044)	0.014 (0.044)	0.028 (0.044)	0.050 (0.045)	0.054 (0.045)	0.027 (0.044)
PF	0.016 (0.011)	0.013 (0.011)	0.017 (0.010)*	0.013 (0.011)	0.019 (0.011)*	0.016 (0.011)
γ_1_	0.029	0.018	0.027	0.087	0.152	0.161
γ_2_	0.296	0.444	0.133	0.209	0.317	0.395
R^2^	0.557	0.610	0.620	0.541	0.643	0.535

The standard error of coefficient estimation is shown in brackets, ‘*’, ‘**’, ‘***’ represent the significance levels of 10%, 5% and 1%, respectively.

In Model 1, when MDPS ≤ 0.029, the application of MDPS has not yet formed scale effect due to the high application cost (Internal Diseconomies) and greenhouse gas emissions (External Diseconomies) in mechanical operations, which results in a negative impact on GTFPG. When 0.029< MDPS ≤ 0.296, economies of scale and environmental benefits gradually emerge (**Table 1**), thereby effectively increasing GTFPG. However, MDPS is heavy-duty mechanical operation and is limited by soil texture. Therefore, overuse of MDPS does not improve GTFPG (He et al., 2021), which can explain why the positive effect on GTFPG is no longer significant when MDPS > 0.296.

The impacts of PSQS are similar to that of MDPS (Model 2). The high application cost of PSQS in the initial development stage decreases GTFPG due to its Diseconomies of Scale. When 0.018< PSQS ≤ 0.444, promotion of PSQS improves GTFPG by increasing seed efficiency, reducing herbicide use, and stabilizing crop yields (Karayel, 2009; Li et al., 2015; Chen et al., 2022). However, PSQS has high requirements for production conditions, technology and management (CPGPRC, 2007). Overuse of PSQS will bring more emissions and wastes, so its positive impact is no longer significant when PSQS > 0.444.

MNTS only has negative impacts on GTFPG no matter how the adoption rate changes (Model 3). The application of MNTS is not yet mature, because of seed quality, mechanical configuration and operation technology, in which China lags behind the developed countries. Large-scale application of MNTS leads to a cost surge, production decline and resources waste (Pisante et al., 2014; Wang et al., 2021b), and causes serious External Diseconomies.

MSRE has a significant positive impact on GTFPG only in the range of 0.087-0.209 (Model 4). In the initial stage, MSRE application has prominent Internal Diseconomies (Jin et al., 2020; Aguiar et al., 2021), thereby significantly reducing GTFPG. In the ecological efficiency stage, ecological effect of MSRE application gradually emerges, thus significantly improving GTFPG. The overuse of MSRE leads to Misallocations and resource waste, which is not conducive to the GTFPG improvement.

The positive effect of MFDD on GTFPG changes from insignificant to significant in different threshold intervals (Model 5). The threshold effect of MFDD on GTFPG shows that the Learning Effects and Facilitation Effects are more prominent, and the External Diseconomies are not significant, so continuous increase of MFDD adoption rate is effective for the improvement of GTFPG.

The positive impacts of WSIR on GTFPG change from insignificant to significant, and then to insignificant (Model 6). The Diseconomies of Scale significantly increases costs and emissions (Moinet et al., 2017), which difficult to increase GTFPG in the initial development stages. When 0.161< WSIR ≤ 0.395, the ecological effects appear, which promote the GTFPG growth. High cost of application makes it difficult to promote WSIR (Zhuang et al., 2019). Recent outflow of rural labor force has led to a decline in rural collective action, which is not conducive to the facility maintenance and technical renewal (Wang et al., 2022). Therefore, its positive impact is no longer significant when WSIR > 0.395.

#### 3.2.2 Threshold analysis of GTFPG in the major grain producing areas

MDPS has the greatest positive impact (1.882), and MNTS has the greatest negative impact (-1.309). Compared with the threshold results of the whole region, the positive impacts of PSQS, MNTS and MSRE on GTFPG in the major grain producing areas are significantly improved, while the positive impacts of MDPS, MFDD and WSIR are not significantly increased (**Table 2**).

**Table 2:** The threshold model results of GTFPG in the major grain producing areas.

	Model 7	Model 8	Model 9	Model 10	Model 11	Model 12
MDPS≤γ_1_	1.882 (1.139)*					
γ_1_<MDPS≤γ_2_	0.701 (0.250)***					
MDPS>γ_2_	0.290 (0.191)					
PSQS≤γ_1_		3.207 (2.427)				
γ_1_<PSQS≤γ_2_		1.029 (0.508)**				
PSQS>γ_2_		0.320 (0.425)				
MNTS≤γ_1_			0.581 (0.285)**			
γ_1_<MNTS≤γ_2_			-1.309 (0.487)***			
MNTS>γ_2_			-0.076 (0.068)			
MSRE≤γ_1_				-0.040 (0.133)		
γ_1_<MSRE≤γ_2_				—		
MSRE>γ_2_				0.388 (0.193)**		
MFDD≤γ_1_					0.045 (0.045)	
γ_1_<MFDD≤γ_2_					—	
MFDD>γ_2_					0.184 (0.054)***	
WSIR≤γ_1_						-0.045 (0.108)
γ_1_<WSIR≤γ_2_						0.140 (0.061)**
WSIR>γ_2_						0.052 (0.058)
Control variables	Control	Control	Control	Control	Control	Control
γ_1_	0.091	0.050	0.172	0.114	0.162	0.195
γ_2_	0.289	0.264	0.339	—	—	0.466
R^2^	0.627	0.471	0.610	0.436	0.468	0.573

The standard error of coefficient estimation is shown in brackets, ‘*’, ‘**’, ‘***’ represent the significance levels of 10%, 5% and 1%, respectively; “—” represent no data.

MDPS can have a great positive impact on GTFPG in the initial development stage (MDPS ≤ 0.289), but the positive impact decreases with the increase of its adoption rate (Model 7). It shows that the mismatch between agricultural mechanization and grain productivity exists even in the major grain producing areas (He et al., 2021).

When 0.050< PSQS ≤ 0.264, its positive effect on GTFPG is remarkably higher than that in the whole region (Model 8). This reflects the importance of the promotion and adoption of PSQS in the major grain producing areas for the improvement of GTFPG.

It has a significant positive impact on GTFPG when MNTS ≤ 0.172 (Model 9). Compared with the whole region results, the MNTS application in the major grain producing areas can have a significant positive impact on GTFPG in a longer period.

When MSRE > 0.114, it has a significant positive impact on GTFPG (Model 10). The increase of MSRE adoption rate doesn’t lead to a negative impact on GTFPG, reflecting that the development of MSRE in the major grain producing areas provides continuous impetus for the improvement of GTFPG.

It has a significant positive impact on GTFPG when MFDD > 0.162. This positive impact is remarkably higher than that in the whole region, reflecting that the application of MFDD has a greater Promoting Effects on GTFPG in the major grain producing areas (Model 11).

When 0.195< WSIR ≤ 0.466, it has a significant positive impact on GTFPG (Model 12). Although the positive impact of WSIR on GTFPG is not significantly improved, the application of this technology plays an important role in improving GTFPG in terms of the impact levels and the threshold value ranges, in both major and non-major producing areas.

#### 3.2.3 Threshold analysis of GTFPG in the non-major grain producing areas

In non-major grain production areas, all green technologies have double threshold impact on GTFPG except MDPS (**Table 3**). WSIR has the greatest positive impact and MFDD the greatest negative impact.

**Table 3:** The threshold model results of GTFPG in the non-major grain producing areas.

	Model 13	Model 14	Model 15	Model 16	Model 17	Model 18
MDPS≤γ_1_	-0.714 (0.273)***					
γ_1_<MDPS≤γ_2_	—					
MDPS>γ_2_	0.341 (0.153)**					
PSQS≤γ_1_		0.044 (0.059)				
γ_1_<PSQS≤γ_2_		0.067 (0.031)**				
PSQS>γ_2_		-0.052 (0.061)				
MNTS≤γ_1_			-0.381 (0.613)			
γ_1_<MNTS≤γ_2_			0.582 (0.283)**			
MNTS>γ_2_			0.025 (0.113)			
MSRE≤γ_1_				-0.584 (0.202)***		
γ_1_<MSRE≤γ_2_				0.491 (0.239)**		
MSRE>γ_2_				0.133 (0.064)		
MFDD≤γ_1_					-1.308 (0.537)**	
γ_1_<MFDD≤γ_2_					0.260 (0.075)***	
MFDD>γ_2_					0.089 (0.135)	
WSIR≤γ_1_						0.631 (0.246)***
γ_1_<WSIR≤γ_2_						0.131 (0.073)*
WSIR>γ_2_						-0.024 (0.052)
Control variables	Control	Control	Control	Control	Control	Control
γ_1_	0.068	0.101	0.055	0.093	0.036	0.079
γ_2_	—	0.459	0.250	0.206	0.295	0.299
R^2^	0.379	0.345	0.494	0.475	0.526	0.464

The standard error of coefficient estimation is shown in brackets, ‘*’, ‘**’, ‘***’ represent the significance levels of 10%, 5% and 1%, respectively; “—” represent no data.

The positive impact of MDPS on GTFPG lasts longer though its impact level is much smaller than that in the whole region and major grain producing areas (Model 13).

The positive impact of PSQS on GTFPG is remarkably smaller than that in the whole region and major grain producing areas, reflecting the limited ability of PSQS to increase GTFPG in the non-major grain producing areas (Model 14).

Compared with the major grain producing areas, the green effect of MNTS application in the non-major grain producing areas is more significant (Model 15), and the increase of MNTS adoption rate in the northern arid and semi-arid non-major grain producing areas is a good illustration.

The positive impact of MSRE on GTFPG is remarkably greater than that in the major grain producing areas (Model 16), meaning that the development and application of MSRE in the non-major grain producing areas has significantly improved GTFPG.

The positive effect is remarkably higher than that in the major grain producing areas, indicating that the application of MFDD in the non-major grain producing areas makes grain production more sustainable (Model 17).

With the increase of WSIR adoption rate, the positive effect gradually decreases (Model 18). It reflects that the application of WSIR is of great significance to improve sustainable development of grain in the non-major grain producing areas, and the agglomeration of provinces with high WSIR adoption rate to some non-major grain producing areas is a good illustration.

### 3.3 Threshold analysis of GECG

#### 3.3.1 Threshold analysis of GECG in the whole region

The threshold results of GECG in the whole region (**Table 4**) shows that MDPS, MSRE, MFDD and WSIR have double threshold effects on GECG, while PSQS and MNTS have single threshold effects on GECG. Among them, MFDD has the greatest positive effect (1.025), and MNTS has the greatest negative effect (-0.793). In Model 19, the positive impact of MDPS on GECG gradually decreases with increasing application. When PSQS > 0.120, it has an insignificant positive impact on GECG, reflecting that PSQS can’t significantly increase GTFPG by affecting GECG (Model 20). Similarly, when MNTS > 0.096, it has an insignificant positive impact on GECG, which reflects that MNTS has no significant effect on GTFPG by affecting GECG (Model 21). MSRE has only a negative impact on GECG, indicating that MSRE decreases GTFPG by affecting GECG (Model 22). When 0.032< MFDD ≤ 0.122, it has the greatest positive impact on GECG, and when MFDD > 0.122, the impact is no longer significant. It reflects that MFDD can’t continue to improve GTFPG through GECG with increasing application (Model 23). Likewise, the threshold results of WSIR for GECG also reflect that as WSIR applications increases, the approach of improving GTFPG by affecting GECG is no longer effective (Model 24).

**Table 4:** The threshold model results of GECG in the whole region.

	Model 19	Model 20	Model 21	Model 22	Model 23	Model 24
MDPS≤γ_1_	0.498 (0.212)**					
γ_1_<MDPS≤γ_2_	0.193 (0.065)***					
MDPS>γ_2_	0.026 (0.021)					
PSQS≤γ_1_		-0.374 (0.180)**				
γ_1_<PSQS≤γ_2_		—				
PSQS>γ_2_		0.032 (0.022)				
MNTS≤γ_1_			-0.793 (0.315)***			
γ_1_<MNTS≤γ_2_			—			
MNTS>γ_2_			0.051 (0.043)			
MSRE≤γ_1_				-0.091 (0.133)		
γ_1_<MSRE≤γ_2_				-0.465 (0.153)***		
MSRE>γ_2_				-0.004 (0.024)		
MFDD≤γ_1_					-0.073 (1.379)	
γ_1_<MFDD≤γ_2_					1.025 (0.376)***	
MFDD>γ_2_					0.028 (0.019)	
WSIR≤γ_1_						-0.455 (0.168)***
γ_1_<WSIR≤γ_2_						0.169 (0.061)***
WSIR>γ_2_						-0.020 (0.024)
UR	0.013 (0.045)	0.069 (0.044)	-0.039 (0.044)	0.010 (0.046)	-0.017 (0.024)	0.015 (0.044)
AM	-0.012 (0.005)***	-0.028 (0.012)	-0.011 (0.013)	-0.006 (0.011)	-0.019 (0.014)	-0.011 (0.011)
IR	0.049 (0.015)***	0.048 (0.014)***	0.081 (0.044)*	0.013 (0.014)	0.012 (0.014)	0.045 (0.018)***
PS	-0.028 (0.034)	-0.013 (0.044)	-0.017 (0.031)	-0.029 (0.029)	-0.023 (0.013)*	-0.011 (0.035)
AF	-0.091 (0.104)	-0.069 (0.124)	-0.046 (0.140)	-0.033 (0.019)*	-0.085 (0.014)	-0.033 (0.014)
AI	0.029 (0.119)	0.050 (0.041)	0.059 (0.031)*	0.082 (0.113)	0.085 (0.117)	0.060 (0.033)*
RI	0.011 (0.054)	0.030 (0.063)	-0.070 (0.052)	-0.050 (0.053)	-0.021 (0.057)	0.006 (0.052)
PA	-0.019 (0.011)*	-0.018 (0.011)*	-0.013 (0.011)	-0.085 (0.031)***	-0.053 (0.026)**	-0.069 (0.012)
TD	-0.045 (0.117)	-0.046 (0.101)	-0.021 (0.061)	-0.058 (0.101)	-0.028 (0.016)*	-0.074 (0.111)
DI	-0.028 (0.016)*	-0.029 (0.015)**	-0.023 (0.013)*	-0.021 (0.016)	-0.024 (0.014)*	-0.021 (0.012)*
TF	0.015 (0.039)	0.017 (0.039)	0.015 (0.039)	0.051 (0.041)	0.014 (0.039)	0.008 (0.039)
PF	0.016 (0.009)*	0.014 (0.008)*	0.016 (0.009)*	0.015 (0.099)	0.016 (0.009)*	0.014 (0.009)
γ_1_	0.067	0.120	0.096	0.109	0.032	0.067
γ_2_	0.346	—	—	0.386	0.122	0.146
R^2^	0.421	0.325	0.366	0.430	0.396	0.448

The standard error of coefficient estimation is shown in brackets, ‘*’, ‘**’, ‘***’ represent the significance levels of 10%, 5% and 1%, respectively; “—” represent no data.

#### 3.3.2 Threshold analysis of GECG in the major grain producing areas

The threshold results of GECG in the major grain producing areas (**Table 5**) show that MDPS, MSRE, MFDD and WSIR have double threshold effects on GECG, while PSQS and MNTS have single threshold effects on GECG. In the major grain producing areas, the positive impact of MDPS on GECG is remarkably higher than that in the whole region. PSQS only has a negative impact on GECG, and the impact is greater than that in the whole region. In the initial development stage (MNTS ≤ 0.085), MNTS can have a great positive impact on GECG, but in other stages (MNTS > 0.085), the positive impact is not significant. With the increasing application of MSRE, its positive impact on GECG gradually decreases, but the overall impact is significantly higher than that in the whole region. The positive impact of both MFDD and WSIR on GECG in ecological efficiency stage is greater than that in the whole region.

**Table 5:** The threshold model results of GECG in the major grain producing areas.

	Model 25	Model 26	Model 27	Model 28	Model 29	Model 30
MDPS≤γ_1_	0.753 (0.117)					
γ_1_<MDPS≤γ_2_	0.353 (0.124)***					
MDPS>γ_2_	-0.059 (0.044)					
PSQS≤γ_1_		-1.495 (0.442)***				
γ_1_<PSQS≤γ_2_		—				
PSQS>γ_2_		-0.022 (0.082)				
MNTS≤γ_1_			6.551 (2.253)***			
γ_1_<MNTS≤γ_2_			—			
MNTS>γ_2_			0.091 (0.074)			
MSRE≤γ_1_				0.299 (0.141)**		
γ_1_<MSRE≤γ_2_				0.099 (0.050)**		
MSRE>γ_2_				0.071 (0.046)		
MFDD≤γ_1_					0.089 (0.184)	
γ_1_<MFDD≤γ_2_					1.167 (0.582)**	
MFDD>γ_2_					0.059 (0.112)	
WSIR≤γ_1_						-0.019 (0.135)
γ_1_<WSIR≤γ_2_						0.270 (0.131)**
WSIR>γ_2_						0.058 (0.085)
Control variables	Control	Control	Control	Control	Control	Control
γ_1_	0.117	0.103	0.085	0.121	0.152	0.046
γ_2_	0.375	—	—	0.513	0.425	0.194
R^2^	0.563	0.484	0.463	0.442	0.441	0.562

The standard error of coefficient estimation is shown in brackets, ‘**’, ‘***’ represent the significance levels of 5% and 1%, respectively; “—” represent no data.

#### 3.3.3 Threshold analysis of GECG in the non-major grain producing areas

The threshold results of GECG in the non-major grain producing areas (**Table 6**) show that MDPS, PSQS, MSRE and WSIR have double threshold effects on GECG, while MNTS and MFDD have single threshold effect on GECG. In the non-major grain producing areas, the positive effects of MDPS, MNTS and WSIR on GECG are smaller than those in the whole region and major grain producing areas, indicating that these technologies have poor effects on improving GTFPG through GECG in the non-major grain producing areas. Besides, the positive impact of PSQS and MFDD on GECG is greater than that in the whole region and major grain producing areas, reflecting that PSQS and MFDD can better improve GTFPG through GECG in the non-major grain producing areas. MSRE has only a negative impact on GECG, and the impact is greater than that in the whole region, indicating that MSRE significantly reduced GTFPG through GECG in the non-major grain producing areas.

**Table 6:** The threshold model results of GECG in the non-major grain producing areas.

	Model 31	Model 32	Model 33	Model 34	Model 35	Model 36
MDPS≤γ_1_	0.062 (0.103)					
γ_1_<MDPS≤γ_2_	0.188 (0.108)*					
MDPS>γ_2_	-0.066 (0.079)					
PSQS≤γ_1_		6.142 (5.528)				
γ_1_<PSQS≤γ_2_		0.237 (0.133)*				
PSQS>γ_2_		0.018 (0.091)				
MNTS≤γ_1_			-1.422 (0.810)*			
γ_1_<MNTS≤γ_2_			—			
MNTS>γ_2_			0.086 (0.218)			
MSRE≤γ_1_				-0.833 (0.242)***		
γ_1_<MSRE≤γ_2_				-0.248 (0.122)**		
MSRE>γ_2_				-0.093 (0.078)		
MFDD≤γ_1_					5.889 (2.221)***	
γ_1_<MFDD≤γ_2_					—	
MFDD>γ_2_					0.039 (0.067)	
WSIR≤γ_1_						-0.820 (0.348)**
γ_1_<WSIR≤γ_2_						-0.301 (0.135)**
WSIR>γ_2_						0.131 (0.168)
Control variables	Control	Control	Control	Control	Control	Control
γ_1_	0.122	0.106	0.123	0.086	0.122	0.067
γ_2_	0.414	0.274	—	0.142	—	0.144
R^2^	0.453	0.463	0.441	0.496	0.480	0.464

The standard error of coefficient estimation is shown in brackets, ‘*’, ‘**’, ‘***’ represent the significance levels of 10%, 5% and 1%, respectively; “—” represent no data.

### 3.4 Threshold analysis of GTCG

#### 3.4.1 Threshold analysis of GTCG in the whole region

The threshold results of GTCG in the whole region shows that only MFDD has a double threshold effect on GTCG, while MDPS, MNTS, MSRE and WSIR have single threshold effects (**Table 7**). PSQS does not have a threshold effect. PSQS has the greatest positive effect on GTCG (0.239), while MSRE has the greatest negative effect (-1.587). In Model 37, the application of MDPS has a negative impact on GTCG, and the negative impact becomes more remarkable with increasing application. Compared with the effect of PSQS on GECG, PSQS mainly improves GTFPG through GTCG (Model 38). MNTS can significantly increase GTCG only in the initial development stage. Increase of MNTS adoption rate hinders the improvement of GTCG (Model 39). In Model 40, the impact of MSRE on GTCG changed from a significant negative impact to significant positive in different threshold intervals, indicating that the large-scale application of MSRE could effectively improve GTCG. This is also why MSRE can improve GEFPG significantly when 0.087< MSRE ≤ 0.209 in Model 4. When MFDD >0.064, the GTCG level is significantly improved, reflecting that increasing application of MFDD can improve GTFPG through GTCG (Model 41). The threshold results of WSIR on GTCG reflect that the increase of WSIR applications improves GTFPG through GTCG (Model 42).

**Table 7:** The threshold model results of GTCG in the whole region.

	Model 37	Model 38	Model 39	Model 40	Model 41	Model 42
MDPS≤γ_1_	-0.451 (0.488)					
γ_1_<MDPS≤γ_2_	—					
MDPS>γ_2_	-1.364 (0.509)***					
PSQS≤γ_1_		—				
γ_1_<PSQS≤γ_2_		—				
PSQS>γ_2_		0.239 (0.124)*				
MNTS≤γ_1_			0.201 (0.116)*			
γ_1_<MNTS≤γ_2_			—			
MNTS>γ_2_			-0.017 (0.051)			
MSRE≤γ_1_				-1.587 (0.787)**		
γ_1_<MSRE≤γ_2_				—		
MSRE>γ_2_				0.161 (0.026)***		
MFDD≤γ_1_					-0.763 (0.295)***	
γ_1_<MFDD≤γ_2_					0.129 (0.043)***	
MFDD>γ_2_					0.042 (0.021)**	
WSIR≤γ_1_						0.097 (0.075)
γ_1_<WSIR≤γ_2_						—
WSIR>γ_2_						0.152 (0.073)**
UR	0.021 (0.050)	0.026 (0.049)	0.031 (0.049)	0.055 (0.031)*	0.035 (0.049)	0.024 (0.049)
AM	0.014 (0.012)	0.013 (0.013)	0.018 (0.011)*	0.027 (0.011)***	0.023 (0.016)	0.016 (0.009)*
IR	0.013 (0.017)	0.069 (0.105)	0.125 (0.051)***	0.012 (0.016)	0.018 (0.011)*	-0.003 (0.020)
PS	0.014 (0.037)	0.007 (0.017)	0.007 (0.036)	-0.055 (0.032)*	0.037 (0.041)	-0.014 (0.009)
AF	0.038 (0.016)***	0.021 (0.012)*	0.085 (0.019)***	0.004 (0.015)	0.031 (0.017)*	0.034 (0.016)**
AI	0.192 (0.113)*	0.172 (0.110)*	0.044 (0.124)	0.034 (0.124)	0.140 (0.068)*	0.028 (0.015)*
RI	-0.013 (0.059)	-0.035 (0.068)	-0.013 (0.027)	-0.017 (0.059)	0.044 (0.026)*	-0.008 (0.058)
PA	-0.005 (0.011)	-0.019 (0.011)*	-0.012 (0.013)	-0.072 (0.124)	-0.070 (0.111)	-0.071 (0.013)
TD	-0.075 (0.110)	-0.012 (0.013)	-0.076 (0.102)	-0.099 (0.130)	-0.031 (0.136)	-0.058 (0.013)
DI	-0.056 (0.018)***	-0.052 (0.018)***	-0.057 (0.018)***	-0.056 (0.017)***	-0.054 (0.018)***	-0.050 (0.018)***
TF	0.011 (0.043)	0.008 (0.043)	0.015 (0.044)	0.029 (0.044)	0.032 (0.044)	0.013 (0.044)
PF	0.006 (0.010)	-0.007 (0.010)	0.006 (0.011)	0.007 (0.011)	0.007 (0.011)	0.004 (0.010)
γ_1_	0.095	—	0.081	0.025	0.064	0.141
γ_2_	—	—	—	—	0.377	—
R^2^	0.345	0.490	0.401	0.344	0.465	0.309

The standard error of coefficient estimation is shown in brackets, ‘*’, ‘**’, ‘***’ represent the significance levels of 10%, 5% and 1%, respectively; “—” represent no data.

#### 3.4.2 Threshold analysis of GTCG in the major grain producing areas

The threshold results of GTCG in the major grain producing areas (**Table 8**) show that only the impact of MDPS on GTCG has single threshold effect, and that of other green technologies has double threshold effect. In the major grain producing areas, the positive impact of MDPS on GTCG is remarkably higher than that in the whole region. PSQS has a positive impact on GTCG, but the impact is smaller than that in the whole region. When MNTS > 0.090, its impact on GTCG is significantly negative and the negative effect is higher than that in the whole region. With increasing application of MSRE, its positive impact on GTCG gradually decreases, and eventually turns to the insignificant negative impact, but the overall impact levels are higher than that in the whole region. With increasing application of MFDD, its positive impact on GTCG gradually decreases and eventually becomes an insignificant negative impact. The impact of WSIR on GTCG in the major grain producing areas is negative in all threshold intervals.

**Table 8:** The threshold model results of GTCG in the major grain producing areas.

	Model 43	Model 44	Model 45	Model 46	Model 47	Model 48
MDPS≤γ_1_	4.307 (1.542)***					
γ_1_<MDPS≤γ_2_	—					
MDPS>γ_2_	-0.029 (0.048)					
PSQS≤γ_1_		1.279 (0.933)				
γ_1_<PSQS≤γ_2_		0.109 (0.051)**				
PSQS>γ_2_		0.028 (0.094)				
MNTS≤γ_1_			2.542 (5.026)			
γ_1_<MNTS≤γ_2_			-7.663 (2.346)***			
MNTS>γ_2_			-0.131 (0.076)*			
MSRE≤γ_1_				1.193 (0.493)**		
γ_1_<MSRE≤γ_2_				0.104 (0.059)*		
MSRE>γ_2_				-0.064 (0.144)		
MFDD≤γ_1_					1.112 (0.443)**	
γ_1_<MFDD≤γ_2_					0.292 (0.174)*	
MFDD>γ_2_					-0.092 (0.120)	
WSIR≤γ_1_						-4.718 (1.326)
γ_1_<WSIR≤γ_2_						-0.501 (0.247)
WSIR>γ_2_						-0.021 (0.092)
Control variables	Control	Control	Control	Control	Control	Control
γ_1_	0.117	0.103	0.090	0.037	0.049	0.093
γ_2_	—	0.444	0.145	0.352	0.371	0.176
R^2^	0.335	0.329	0.347	0.344	0.368	0.351

The standard error of coefficient estimation is shown in brackets, ‘*’, ‘**’, ‘***’ represent the significance levels of 10%, 5% and 1%, respectively; “—” represent no data.

#### 3.4.3 Threshold analysis of GTCG in the non-major grain producing areas

The threshold results of GTCG in the non-major grain producing areas (**Table 9**) show that the impact of PSQS and MFDD on GTCG has double threshold effect, and the impact of other green technologies has single threshold effect. In the non-major grain producing areas, MDPS has a negative impact on GTCG, and the impact is especially significant when MDPS ≤ 0.178. Compared with that in the whole region and major grain producing areas, the impact of PSQS on GTCG is positive only when it is smaller than the first threshold value. MNTS, MSRE, MFDD and WSIR have a positive impact on GTCG, and the positive impact of MFDD and WSIR is higher than that in the whole region and the major grain producing areas.

**Table 9:** The threshold model results of GTCG in the non-major grain producing areas.

	Model 49	Model 50	Model 51	Model 52	Model 53	Model 54
MDPS≤γ_1_	-4.386 (2.017)**					
γ_1_<MDPS≤γ_2_	—					
MDPS>γ_2_	-0.024 (0.096)					
PSQS≤γ_1_		1.446 (0.308)***				
γ_1_<PSQS≤γ_2_		-0.221 (0.119)*				
PSQS>γ_2_		-0.016 (0.110)				
MNTS≤γ_1_			0.611 (0.287)**			
γ_1_<MNTS≤γ_2_			—			
MNTS>γ_2_			0.084 (0.196)			
MSRE≤γ_1_				0.286 (0.142)**		
γ_1_<MSRE≤γ_2_				—		
MSRE>γ_2_				0.082 (0.095)		
MFDD≤γ_1_					1.688 (0.737)**	
γ_1_<MFDD≤γ_2_					0.254 (0.126)**	
MFDD>γ_2_					0.055 (0.083)	
WSIR≤γ_1_						0.041 (0.082)
γ_1_<WSIR≤γ_2_						—
WSIR>γ_2_						0.374 (0.185)**
Control variables	Control	Control	Control	Control	Control	Control
γ_1_	0.178	0.104	0.182	0.134	0.036	0.143
γ_2_	—	0.273	—	—	0.291	—
R^2^	0.427	0.452	0.424	0.429	0.412	0.491

The standard error of coefficient estimation is shown in brackets, ‘*’, ‘**’, ‘***’ represent the significance levels of 10%, 5% and 1%, respectively; “—” represent no data.

## 4 Discussions

The concept of major grain producing areas has attracted more resources to the provinces in these areas, and has profoundly affected the behavior and decision-making of producers (SCPRC, 2017; Yang et al., 2021). Besides, the resource wastes and environmental pressures caused by the continuous growth of grain production in major producing areas (Yang et al., 2021; Li and Lin, 2022), arouse more attention to the coordinated use of various green technologies. Additionally, the influence paths of green technology on the promotion of GTFPG also vary in different areas, due to regional differences in development and applicability of green technologies (Si et al., 2021; He et al., 2021).

### 4.1 Green technologies in plowing

The adoption rate of MDPS in the major grain producing areas is higher, especially in Northwest, Northeast and Yellow River production areas, reflecting that the major grain producing areas pay more attention to the green technology application in plowing.

During the ecological efficiency stage of MDPS, the ecological effects of technology application in the major grain producing areas are greater than those in the non-major grain producing areas, which is consistent with He et al. (2021). Moreover, there is overuse of MDPS in the major grain producing areas, which may be a result of resource Misallocations caused by Path Dependences or External Diseconomies (Li et al., 2017; Xie et al., 2021). However, there is no overuse of MDPS in the non-major grain producing areas, which may be related to its advantages in mechanization, proficiency of technical staff and social services (especially provinces with higher level of economic development).

In the major grain producing areas, MDPS mainly affects GTFPG through the GTCG path in the initial development stage, which indicates that the Spillover Effects are more significant in this period; MDPS improves GTFPG mainly through the GECG path in the ecological efficiency stage, which may be related to the Learning Effects, Synergy Effects and Promoting Effects generated by the MDPS application (**Figure 1**). In the non-major grain producing areas, MDPS increases GTFPG mainly through the GECG path. The green technology efficiency change caused by the MDPS application is more prominent in the major grain producing areas. However, the contribution of MDPS to green technical progress change is very limited, and the Diseconomies of Scale caused by technological upgrading are especially serious in the non-major grain producing areas.

### 4.2 Green technologies in sowing

High adoption rate of PSQS agglomerates in Inner Mongolia-Northeast China, Xinjiang and Huang-Huai-Hai area, and high adoption rate of MNTS gathers in Huang-Huai-Hai area, reflecting that the importance attached to green sowing technologies by major grain producing areas.

The difference between PSQS and MNTS threshold results can reflect that the ecological effect of PSQS application is better than that of MNTS, especially in the major grain producing areas, which is consistent with Li et al. (2015) and Chen et al. (2022), and also explains the higher adoption rate of PSQS. The application of MNTS has high requirements for natural conditions, mechanical configuration, seed quality and technical levels (Duan et al., 2022a). Improper application of MNTS brings serious External Diseconomies, which limits its ecological effects and application scopes.

In the major grain producing areas, PSQS mainly improves GTFPG through the GTCG path, which indicates that the Facilitation Effects, Acceleration Actions and Group Identities caused by the PSQS application are more prominent (**Figure 1**); however, MNTS application features high operational risk, long investment return period, and high operational requirements (Zhang et al., 2021; Wang et al., 2021b), which lead to the External Diseconomies, Misallocation and Adaptive Effects, and mainly improve GTFPG through the GTCG path. In the non-major grain producing areas, some economically developed and highly mechanized provinces can support the mature application of PSQS, and are more prominent in the Learning effects, Synergy Effects and Promoting effects, which lead to improve GTFPG through the GECG path. Meanwhile, the arid and semi-arid areas in the non-major grain producing areas are suitable for the promotion and application of MNTS (Zhang et al., 2018), which bring significant Facilitation Effects, Acceleration Actions and Group identities, and mainly improve GTFPG through the GTCG path. This also explains the increase of MNTS adoption rate in Qinghai, Shanxi and Shaanxi in recent years.

### 4.3 Green technologies in fertilization

The adoption rate of MSRE in the major grain producing areas is significantly higher than that in the non-major grain producing areas, while the adoption rate of MFDD in the two areas is similar, which reflects that major grain producing areas pay more attention to the green fertilization technologies.

In the major grain producing areas, MSRE and MFDD can continuously improve GTFPG (the increase in technology adoption rate does not cause a negative threshold effect), and the ecological effect of MSRE is greater. In the non-major grain producing areas, MSRE and MFDD only have significant positive impacts on GTFPG between the first and second threshold values, and the effects are greater than that in the major grain producing areas, and MSRE has more obvious advantages. While, there are overuse of the two green technologies in the non-major grain producing areas.

Application of MSRE requires complex conditions (**Table S1**), and more straw returning brings higher production costs under the intensive production and rotation system (Yang et al., 2020), resulting in significant Path Dependences and Inertial Actions (**Figure 1**). In addition to the imperfect subsidy system (Huang et al., 2019), MSRE can’t continuously increase GECG. Therefore, MSRE improves GTFPG mainly through the GTCG path in both major and non-major grain producing areas. The differences in the influence paths of MFDD in major and non-major grain producing areas are caused by different natural conditions, economic development, technological levels, and green conception. Especially in the ecological efficiency stage of MFDD application, the Learning Effects, Synergy Effects and Promoting Effects of MFDD application in the major grain producing areas are more prominent; while, the Facilitation Effects, Acceleration Actions and Group identities are more prominent in non-major grain producing areas.

### 4.4 Green technologies in irrigation

The adoption rate of WSIR in the non-major grain producing areas is slightly higher than that in the major grain producing areas, but with higher sample dispersion. This indicates that the non-major grain producing areas attach great importance to the development of green irrigation technologies.

The difference in the threshold effects of WSIR reflects the obvious External Diseconomies in the initial stage of WSIR application in the major grain producing areas, which supports the conclusions of Zhuang et al. (2019) and Chen et al. (2022). However, it shows better green effects in ecological efficiency stage, which explains the high adoption rate of WSIR in the major grain producing areas. In the non-major grain producing areas, WSIR has significant ecological effects in the initial stage. It may be related to the water-saving effects of WSIR in water-deficient areas, especially in Xinjiang, Qinghai, Gansu and Shaanxi (Zhuang et al., 2019; Duan et al., 2022b; Guo et al., 2022a). Moreover, the reason why the overuse stage of WSIR is advanced in the non-major grain producing areas may be that the rural labor transfer reduces collective actions, and is not conducive to the maintenance of irrigation facilities and green efficiency improvement, which supporting the conclusions of Wang et al. (2022).

As an important practice of sustainable agricultural production (Zhuang et al., 2019), the application of WSIR has brought significant green efficiency improvement to major grain producing areas (Man et al., 2014; Wang et al., 2020b). Moreover, External Diseconomies, Misallocation and Adaptive Effects limit the contribution of WSIR to the green technology progress in the major grain producing areas. Regions in the non-major grain producing areas vary greatly in precipitation, so choosing the appropriate water-saving irrigation method is the key to achieving green production (Zhuang et al., 2019; Chen et al., 2022). Therefore, the applications and upgrading of various water-saving technologies in the non-major grain producing areas in recent years have enabled WSIR to make a greater contribution to green technology progress in these areas, which supporting the conclusions of Zhuang et al. (2019).

## 5 Conclusions

This paper took the influence mechanism of green technologies on GTFPG as the entry point, selected green technologies from the plowing, sowing, fertilization, and irrigation section in agricultural mechanized production, and constructed threshold models to explore the impacts of various green technologies on GTFPG and the influence paths. The main conclusions are as follows:

(1) GTFPG and green technologies exhibited correlations as well as regional differences in spatial evolution. The difference of GTFPG among provinces in China gradually decreased, which was mainly caused by the regional difference of GTCG, while the regional difference of GECG remained small. Major grain producing areas and non-major grain producing areas had different preferences for green technologies. Major grain producing areas paid more attention to the green technologies in plowing, sowing and fertilization; while, the green irrigation technology was more widely used in non-major grain producing areas.

(2) In the major grain producing areas, MDPS had the greatest positive impact on GTFPG. In the non-major grain producing areas, the positive impact of WSIR was greatest. In plowing, MDPS had greater ecological effects in the major grain producing areas than in the non-major grain producing areas; however, the overuse of MDPS occurred in the major grain producing areas, but not in the non-major grain producing areas. In sowing, PSQS had better ecological effects than MNTS, especially in the major grain producing areas; the negative impact of MNTS was more significant in the major grain producing areas. In fertilization, overuse of MSRE and MFDD never occurred in the major grain producing areas; in the ecological efficiency stage, MSRE and MFDD had greater positive impacts on GTFPG in the non-major grain producing areas. In irrigation, WSIR showed better ecological effects in the major grain producing areas, and the negative impacts of its overuse were greater in the non-major grain producing areas.

(3) There were significant differences in the influence paths of green technologies on GTFPG of major grain producing areas and non-major grain producing areas. In the major grain producing areas, MDPS (in the ecological efficiency stage), MFDD (in the ecological efficiency stage) and WSIR mainly improved GTFPG through the GECG path; MDPS (in the initial development stage), PSQS, MNTS, MSRE (in the initial development and ecological efficiency stage), and MFDD (in the initial development stage) mainly affected GTFPG through the GTCG path. In the non-major grain production areas, MDPS, PSQS and MFDD (in the initial development stage) increased GTFPG mainly through the GECG path; MNTS, MSRE, MFDD (in the ecological efficiency stage) and WSIR mainly improved GTFPG through the GTCG path.

## Data availability statement

The raw data supporting the conclusions of this article will be made available by the authors, without undue reservation.

## Author contributions

JL: Conceptualization, methodology, software, validation, investigation, resources, data analysis, writing-original draft preparation, writing-review and editing, visualization, funding acquisition. QL: Conceptualization, software, writing-review and editing, supervision, validation, project administration. All authors contributed to the article and approved the submitted version.

## References

[B1] Aguiar A. Milessi T. S. Mulinari D. R. Lopes M. S. da Costa S. M. Candido R. G. (2021). Sugarcane straw as a potential second generation feedstock for biorefinery and white biotechnology applications. Biomass Bioenerg. 144, 105896. doi: 10.1016/j.biombioe.2020.105896

[B2] Baležentis T. Blancard S. Shen Z. Štreimikienė D. (2021). Analysis of environmental total factor productivity evolution in european agricultural sector. Decis. Sci. 52 (2), 483–511. doi: 10.1111/deci.12421

[B3] Baumhardt R. L. Jones O. R. Schwartz R. C. (2008). Long-term effects of profile-modifying deep plowing on soil properties and crop yield. Soil Sci. Soc. America J. 72 (3), 677–682. doi: 10.2136/sssaj2007.0122

[B4] Chaudhary V. Chandra R. Chaudhary R. Bhattacharyya R. (2021). Global warming potential and energy dynamics of conservation tillage practices for different rabi crops in the indo-gangetic plains. J. Environ. Manage. 296, 113182. doi: 10.1016/j.jenvman.2021.113182 34229138

[B5] Chen M. Chen J. Lai S. (2006). Inventory analysis and spatial feature recognition of agricultural and rural pollution in China. China Environ. Sci. 6, 751–755. doi: 10.1016/S0379-4172(06)60102-9

[B6] Cheng G. (2014). Data envelopment analysis method and MaxDEA software (Beijing: Intellectual Property Publishing House).

[B7] Chen Y. Miao J. Zhu Z. (2021). Measuring green total factor productivity of china’s agricultural sector: A three-stage SBM-DEA model with non-point source pollution and CO_2_ emissions. J. Clean. Prod. 318, 128543. doi: 10.1016/j.jclepro.2021.128543

[B8] Chen L. Xin J. Liu J. Yuan M. Liu S. Jiang W.. (2017). Changes in bacterial community of soil induced by long-term straw returning. Sci. Agric. 74, 349–356. doi: 10.1590/1678-992X-2016-0025

[B9] Chen M. Xue W. Chen J. (2022). Platform subsidy policy design for green product diffusion. J. Clean. Prod. 359, 132039. doi: 10.1016/j.jclepro.2022.132039

[B10] Chen J. Pang D. Jin M. Luo Y. Li H. Li Y.. (2020). Improved soil characteristics in the deeper plough layer can increase grain yield of winter wheat. J. Integr. Agriculture. 19 (5), 1215–1226. doi: 10.1016/S2095-3119(19)62679-1

[B11] Clapp J. Moseley W. G. Burlingame B. Termine P. (2022). Viewpoint: The case for a six-dimensional food security framework. Food Policy. 106, 102164. doi: 10.1016/j.foodpol.2021.102164

[B12] CPGPRC. (2007). Agricultural science popularization: high-yield cultivation technology of wheat precision and small quantity sown. Available at: http://www.gov.cn/govweb/fwxx/kp/2007-10/10/content_771816.htm.

[B13] Ding J. Wei H. Yang Y. Zhang J. Wu J. (2018). Effects of conservation tillage on soil water condition and winter wheat yield in farmland. Chin. J. Appl. Ecol. 29, 2501–2508. doi: 10.13287/j.1001-9332.201808.005 30182588

[B14] Duan P. Liu R. Chen S. D. (2022b). Payment decisions on water-saving irrigation services and farming households’incomes: Based on survey data in the ecologically fragile areas of xinjiang, China. Resour. Sci. 44 (4), 833–846. doi: 10.18402/resci.2022.04.15

[B15] Duan Y. Wu M. LV J. Xiang W. Yan B. Ma L.. (2022a). Research status and development suggestions of no-tillage seeding anti-blocking technology. J. Agric. Sci. Technol. 24 (2), 124–135. doi: 10.13304/j.nykjdb.2021.0370

[B16] Eanes F. R. Singh A. S. Bulla B. R. Ranjan P. Fales M. Wickerham B.. (2019). Crop advisers as conservation intermediaries: perceptions and policy implications for relying on nontraditional partners to increase U.S. farmers’ adoption of soil and water conservation practices. Land Use Policy 81 (10), 360–370. doi: 10.1016/j.landusepol.2018.10.054

[B17] ECOSOC. (2022). Progress report on the 10 year framework of programmes on sustainable consumption and production patterns. In: United Nations Conference on Sustainable Development, Rio, Brazil. Available at: https://sdgs.un.org/sites/default/files/publications/1444HLPF_10YFP2.pdf.

[B18] Elkington J. (1994). Towards the sustainable corporation: Win-win-win business strategies for sustainable development. California Manage. Rev. 36 (2), 90–100. doi: 10.2307/41165746

[B19] Fabiani S. Vanino S. Nino P. Napoli R. (2020). Water energy food nexus approach for sustainability assessment at farm level: an experience from an intensive agricultural area in central italy. Environ. Sci. Policy 104, 1–12. doi: 10.1016/j.envsci.2019.10.008

[B20] FAO IFAD UNICEF WFP WHO. (2022). “The state of food security and nutrition in the world 2022,” in Repurposing food and agricultural policies to make healthy diets more affordable (Rome: FAO). doi: 10.4060/cc0639en

[B21] Frank S. Havlík P. Stehfest E. van Meijl H. Witzke P. Perez-Domínguez I.. (2019). Agricultural non-CO2 emission reduction potential in the context of the 1.5 0C target. Nat. Climate Change 9, 66e72. doi: 10.1038/s41558-018-0358-8

[B22] FSIN. (2022). Global report on food crises. Available at: https://www.fsinplatform.org/sites/default/files/resources/files/GRFC%202022%20KM%20ENG%20ARTWORK.pdf (Accessed September 12, 2022).

[B23] Gaihre Y. Singh U. Islam S. Huda A. Islam M. Satter M.. (2015). Impacts of urea deep placement on nitrous oxide and nitricoxide emissions from rice fields in bangladesh. Geoderma. 259, 370–379. doi: 10.1016/j.geoderma.2015.06.001

[B24] Gao W. (2022). Research on green innovation driving mechanism for high-quality development of grain industry. J. Jiangxi Univ. Finance Econ. 3, 73–86. doi: 10.13,676/j.cnki.cn36-1224/f.2022.03.002

[B25] Gao L. Zhang W. Mei Y. Abdoul G. Song Y. Jin S. (2018). Do farmers adopt fewer conservation practices on rented land? evidence from straw retention in China. Land Use Policy. 79, 609–621. doi: 10.1016/j.landusepol.2018.08.026

[B26] Guo Z. Chen X. Zhang Y. (2022b). Impact of environmental regulation perception on farmers’ agricultural green production technology adoption: A new perspective of social capital. Technol. Soc. 71, 102085. doi: 10.1016/j.techsoc.2022.102085

[B27] Guo Y. Qiu L. Yao S. (2022a). Analysis of the impact of irrigation water-saving techniques adoption on Farmers’Agricultural income. On Econ. Problems 04), 93–100. doi: 10.16011/j.cnki.jjwt.2022.04.008

[B28] Hansen B. E. (1999). Threshold effects in non-dynamic panels: estimation, testing, and inference. J. Econom. 93 (2), 345–368. doi: 10.1016/S0304-4076(99)00025-1

[B29] He P. Zhang J. Li W. (2021). The role of agricultural green production technologies in improving low-carbon efficiency in china: necessary but not effective. J. Environ. Manage. 293, 112837. doi: 10.1016/j.jenvman.2021.112837 34102495

[B30] Houssou N. Diao X. Cossar F. Kolavalli S. Jimah K. Aboagye P. (2013). Agricultural mechanization in ghana: Is specialized agricultural mechanization service provision a viable business model? Am. J. Agric. Economics 95 (5), 1237–1244. doi: 10.1093/ajae/aat026

[B31] Huang X. Cheng L. Chien H. Jiang H. Yang X. Yin C. (2019). Sustainability of returning wheat straw to field in hebei, Shandong and jiangsu provinces: a contingent valuation method. J. Clean. Prod. 213, 1290–1298. doi: 10.1016/j.jclepro.2018.12.242

[B32] Huisingh D. Zhang Z. Moore J. C. Qiao Q. Li Q. (2015). Recent advances in carbon emissions reduction: policies, technologies, monitoring, assessment and modeling. J. Clean. Prod. 103, 1–12. doi: 10.1016/j.jclepro.2015.04.098

[B33] Hu J. Xue L. Qian C. Xue L. Cao S. (2022). Effects of oxygen enrichment on surface water nutrient dynamics and greenhouse gas emissions in paddy fields with different straw returning. Environ. Sci. doi: 10.13227/j.hjkx.202204079 37040983

[B34] Jiang M. Hu X. Chunga J. Lin Z. Fei R. (2020). Does the popularization of agricultural mechanization improve energy-environment performance in china’s agricultural sector? J. Clean. Prod. 276 (1), 124210. doi: 10.1016/j.jclepro.2020.124210

[B35] Jin L. B. Cui H. Y. Li B. Zhang J. W. Dong S. T. Liu P. (2012). Effects of integrated agronomic management practices on yield and nitrogen efficiency of summer maize in north China. Field Crops Res. 134, 30–35. doi: 10.1016/j.fcr.2012.04.008

[B36] Jin Z. Shah T. Zhang L. Liu H. Peng S. Nie L. (2020). Effect of straw returning on soil organic carbon in rice–wheat rotation system: A review. Food Energy Secur. 9 (2), e200. doi: 10.1002/fes3.200

[B37] Johnes J. (2015). Operational research in education. Eur. J. Oper. Res. 243, 683–696. doi: 10.1016/j.ejor.2014.10.043

[B38] Karayel D. (2009). Performance of a modified precision vacuum seeder for no-till sowing of maize and soybean. Soil tillage Res. 104 (1), 121–125. doi: 10.1016/j.still.2009.02.001

[B39] Keshavarz Afshar R. Dekamin M. (2022). Sustainability assessment of corn production in conventional and conservation tillage systems. J. Clean. Prod. 351, 131508. doi: 10.1016/j.jclepro.2022.131508

[B40] Li Q. Bian C. Liu X. Ma C. Liu Q. (2015). Winter wheat grain yield and water use efficiency in wide-precision planting pattern under deficit irrigation in north China plain. Agric. Water Manage. 153, 71–76. doi: 10.1016/j.agwat.2015.02.004

[B41] Li H. Dai M. Dai S. Dong X. (2018). Current status and environment impact of direct straw return in china’s cropland – a review. Ecotoxicol. Environ. Safety 159, 293–300. doi: 10.1016/j.ecoenv.2018.05.014 29763811

[B42] Li J. Lin Q. (2022). Can the adjustment of china’s grain purchase and storage policy improve its green productivity? Int. J. Environ. Res. Public Health 19, 6310. doi: 10.3390/ijerph19106310 35627849 PMC9140889

[B43] Li T. Long H. Zhang Y. Ge D. Li Y. (2017). Analysis of the spatial mismatch of grain production and farmland resources in China based on the potential crop rotation system. Land Use Policy. 60, 26–36. doi: 10.1016/j.landusepol.2016.10.013

[B44] Li J. Song Z. (2022). Dynamic impacts of external uncertainties on the stability of the food supply chain: Evidence from China. Foods 11, 2552. doi: 10.3390/foods11172552 36076737 PMC9454885

[B45] Liu Y. Feng C. (2019). What drives the fluctuations of “green” productivity in china’s agricultural sector? a weighted Russell directional distance approach. Res. Conserv. Recy. 147, 201–213. doi: 10.1016/j.resconrec.2019.04.013

[B46] Liu Z. Guan D. Crawford-Brown D. Zhang Q. He K. Liu J. (2013). Energy policy: A low-carbon road map for China. Nat. (London) 500 (7461), 143–145. doi: 10.1038/500143a 23925225

[B47] Liu S. Lei P. Li X. Li Y. (2022). A nonseparable undesirable output modified three-stage data envelopment analysis application for evaluation of agricultural green total factor productivity in China. Sci. Total Environ. 838, 155947. doi: 10.1016/j.scitotenv.2022.155947 35577090

[B48] Liu Y. Sun D. Wang H. Wang X. Yu G. Zhao X. (2020). An evaluation of china’s agricultural green production: 1978-2017. J. Clean. Prod. 243, 118483.1–118483.12. doi: 10.1016/j.jclepro.2019.118483

[B49] Li M. Wang J. Zhao P. Chen K. Wu L. (2020). Factors affecting the willingness of agricultural green production from the perspective of farmers’ perceptions. Sci. total environ. 738, 140289. doi: 10.1016/j.scitotenv.2020.140289 32806378

[B50] Luan B. Huang J. Zou H. (2019). Domestic R&D, technology acquisition, technology assimilation and china’s industrial carbon intensity: evidence from a dynamic panel threshold model. Sci. Total Environ. 693, 133436.1–133436.11. doi: 10.1016/j.scitotenv.2019.07.242 31377352

[B51] Maňez J. A. Love J. H. (2020). Quantifying sunk costs and learning effects in R&Dd persistence. Res. Policy. 49, 104004. doi: 10.1016/j.respol.2020.104004

[B52] Man J. Wang D. White P. Yu Z. (2014). The length of micro-sprinkling hoses delivering supplemental irrigation affects photosynthesis and dry matter production of winter wheat. Field Crops Res. 168, 65–74. doi: 10.1016/j.fcr.2014.08.012

[B53] Mao H. Zhou L. Ying R. Pan D. (2021). Time preferences and green agricultural technology adoption: Field evidence from rice farmers in China. Land Use Policy. 109, 105627. doi: 10.1016/j.landusepol.2021.105627

[B54] MARAPRC. (2018). Technical guidelines for agricultural green development(2018-2030). Available at: http://www.moa.gov.cn/govpublic/KJJYS/201807/t20180706_6153629.htm.

[B55] Miah M. Gaihre Y. Hunter G. Singh U. Hossain S. (2016). Fertilizer deep placement increases rice production: evidence from farmers’ fields in southern bangladesh. Agron. J. 108 (2), 805–812. doi: 10.2134/agronj2015.0170

[B56] Midingoyi S. Kassie M. Muriithi B. Diiro G. Ekesi S. (2018). Do farmers and the environment benefit from adopting integrated pest management practices? evidence from Kenya. J. Agric. Econ. 70, 452–470. doi: 10.1111/1477-9552.12306

[B57] Min S. Paudel K. P. Chen F. B. (2021). Mechanization and efficiency in rice production in china. J. Integr. Agri. 20 (7), 1996–2008. doi: 10.1016/S2095-3119(20)63439-6

[B58] Moinet G. Cieraad E. Turnbull M. Whitehead D. (2017). Effects of irrigation and addition of nitrogen fertiliser on net ecosystem carbon balance for a grassland. Sci. Total Environ. 579, 1715e1725. doi: 10.1016/j.scitotenv.2016.11.199 27923580

[B59] Nagothu U. S. (2018). “Summary: sustainable intensification of agriculture, technology and policy options,” in Agricultural development and sustainable intensification (London, New York: Routledge), 274–296.

[B60] Oh D. (2010). A global malmquist-luenberger productivity index. J. Product. Anal. 34, 183–197. doi: 10.1007/s11123-010-0178-y

[B61] Pan X. Wei Z. Han B. Shahbaz M. (2021). The heterogeneous impacts of interregional green technology spillover on energy intensity in china. Energy Econ. 96, 105133. doi: 10.1016/j.eneco.2021.105133

[B62] Peixoto D. S. Silva L. Melo L. B. B. Azevedo R. P. Araujo B. C. L. Carvalho T. S.. (2020). Occasional tillage in no-tillage systems: a global meta-analysis. Sci. total environ. 745, 140887. doi: 10.1016/j.scitotenv.2020.140887 32717599

[B63] Pisante M. Stagnari F. Acutis M. Bindi M. Brilli L. Stefano V. D.. (2014). “Conservation agriculture and climate change,” in Conservation agriculture. Eds. Farooq M. Siddique K. (Switzerland: Springer, Cham).

[B64] Purvis B. Mao Y. Robinson D. (2019). Three pillars of sustainability: in search of conceptual origins. Sustain. Sci. 14 (3), 681–695. doi: 10.1007/s11625-018-0627-5

[B65] Qiu T. Luo B. (2021). Do small farms prefer agricultural mechanization services? evidence from wheat production in china. Appl. Econ. 53 (26), 2962–2973. doi: 10.1080/00036846.2020.1870656

[B66] Rahim H. Ghazali M. Bookeri M. Abu Bakar B. Ariff E. Rahman M.. (2021). Economic potential of rice precision farming in malaysia: The case study of felcra seberang perak. Precis. Agri. 23 (3), 812–829. doi: 10.1007/s11119-021-09862-3

[B67] Rahman H. Haque K. M. S. Khan Z. H. (2021). A review on application of controlled released fertilizers influencing the sustainable agricultural production: A cleaner production process. Environ. Technol. Innov. 23, 101697. doi: 10.1016/j.eti.2021.101697

[B68] Reza-Gharehbagh R. Hafezalkotob A. Makui A. Sayadi M. (2022). Financing green technology development and role of digital platforms: Insourcing vs. outsourcing. Technol. Soc. 69, 101967. doi: 10.1016/j.techsoc.2022.101967

[B69] SCPRC. (2017). Available at: http://www.gov.cn/zhengce/content/2017-02/04/content_5165309.htm.

[B70] Shah S. M. Liu G. Yang Q. Casazza M. Agostinho F. Giannetti B. F.. (2021). Sustainability assessment of agriculture production systems in pakistan: a provincial-scale energy-based evaluation. Ecol. Model. 455, 109654. doi: 10.1016/j.ecolmodel.2021.109654

[B71] Shao Y. Xie Y. Wang C. Yue J. Yao Y. Li X.. (2016). Effects of different soil conservation tillage approaches on soil nutrients, water use and wheat-maize yield in rainfed dry-land regions of north China. Eur. J. Agron. 81, 31–45. doi: 10.1016/j.eja.2016.08.014

[B72] Shen Z. Baležentis T. Chen X. Valdmanis V. (2018). Green growth and structural change in Chinese agricultural sector during 1997–2014. China Econ. Rev. 51, 83–96. doi: 10.1016/j.chieco.2018.04.014

[B73] Shi X. Li L. (2019). Green total factor productivity and its decomposition of Chinese manufacturing based on the MML index:2003–2015. J. Clean. Prod. 222, 998–1008. doi: 10.1016/j.jclepro.2019.03.080

[B74] Si R. Aziz N. Liu M. Lu Q. (2021). Natural disaster shock, risk aversion and corn farmers’ adoption of degradable mulch film: evidence from zhangye, china. Int. J. Climate Change Strat. Manage. 13, 60–77. doi: 10.1108/IJCCSM-08-2020-0090

[B75] Silva-Olaya A. Cerri C. La Scala N. Jr. Dias C. Cerri C. (2013). Carbon dioxide emissions under different soil tillage systems in mechanically harvested sugarcane. Environ. Res. Lett. 8 (1), 1–8. doi: 10.1088/1748-9326/8/1/015014

[B76] Song M. Fisher R. Wang J. Cui L. (2018). Environmental performance evaluation with big data: Theories and methods. Ann. Operations Res. 270 (1–2), 459–472. doi: 10.1007/s10479-016-2158-8

[B77] Sun J. Wang Z. Du Y. Zhang E. Gan H. Sun D.. (2022). Optimized tillage improves yield and energy efficiency while reducing carbon footprint in winter wheat-summer maize rotation systems. Sci. Total Environ. 820, 153278. doi: 10.1016/j.scitotenv.2022.153278 35074378

[B78] Sun M. Ren A. Gao Z. Wang P. Mo F. Xue L.. (2018). Long-term evaluation of tillage methods in fallow season for soil water storage, wheat yield and water use efficiency in semiarid southeast of the loess plateau. Field Crops Res. 218, 24–32. doi: 10.1016/j.fcr.2017.12.021

[B79] Tang D. Shan Z. He J. Zhao Z. (2022). How do environmental regulations and outward foreign direct investment impact the green total factor productivity in China? a mediating effect test based on provincial panel data. Int. J. Environ. Res. Public Health 19, 15717. doi: 10.3390/ijerph192315717 36497791 PMC9740457

[B80] Tang D. Tang J. Xiao Z. Ma T. Bethel B. J. (2017). Environmental regulation efficiency and total factor productivity–effect analysis based on chinese data from 2003 to 2013. Ecol. Indic. 73, 312–318. doi: 10.1016/j.ecolind.2016.08.040

[B81] Tian P. Sui P. Lian H. Wang Z. Meng G. Sun Y.. (2019). Maize straw returning approaches affected straw decomposition and soil carbon and nitrogen storage in northeast china. Agron. (Basel). 9 (12), 818. doi: 10.3390/agronomy9120818

[B82] Tone K. Tsutsui M. (2010). An epsilon-based measure of efficiency in dea-a third pole of technical efficiency. Eur. J. Oper. Res. 207, 1554–1563. doi: 10.1016/j.ejor.2010.07.014

[B83] Totin E. Van Mierlo B. Klerkx L. (2020). Scaling practices within agricultural innovation platforms: Between pushing and pulling. Agric. Syst. 179, 102764. doi: 10.1016/j.agsy.2019.102764

[B84] Tugcu C. Tiwari A. (2016). Does renewable and/or non-renewable energy consumption matter for total factor productivity (TFP) growth? evidence from the BRICS. Renewable Sustain. Energy Rev. 65, 610–616. doi: 10.1016/j.rser.2016.07.016

[B85] UN. (2001). Road map towards the implementation of the united nations millennium declaration. Available at: https://mdgs.un.org/unsd/mdg/Resources/Static/Products/SGReports/56_326/a_56_326e.pdf.

[B86] UN. (2015). Transforming our world: the 2030 agenda for sustainable development. Available at: https://documents-dds-ny.un.org/doc/UNDOC/GEN/N15/291/89/PDF/N1529189.pdf?OpenElement.

[B87] Wang N. (2021). Mechanized technology for deep application of chemical fertilizer to corn in heilongjiang province. New Agric. 14, 66.

[B88] Wang H. Fang L. Mao H. Chen S. (2022). Can e-commerce alleviate agricultural non-point source pollution? — A quasi-natural experiment based on a china’s e-commerce demonstration city. Sci. Total Environ. 846, 157423. doi: 10.1016/j.scitotenv.2022.157423 35853527

[B89] Wang S. Huang X. Zhang Y. Yin C. Richel A. (2021a). The effect of corn straw return on corn production in northeast China: An integrated regional evaluation with meta-analysis and system dynamics. Res. Conserv. Recy. 167, 105402. doi: 10.1016/j.resconrec.2021.105402

[B90] Wang Y. Su Y. Shu Q. (2022). Labor out-migration, rural collective action and rural revitalization. J. Tsinghua University(Philos. Soc. Sci.) 37, 173–187. doi: 10.13613/j.cnki.qhdz.003150

[B91] Wang S. Wang H. Hafeez M. D. Zhang Q. Yu Q. Wang R.. (2020a). No-tillage and subsoiling increased maize yields and soil water storage under varied rainfall distribution: A 9-year site-specific study in a semi-arid environment. Field Crops Res. 255, 107867. doi: 10.1016/j.fcr.2020.107867

[B92] Wang Q. Xu Q. Lu C. Li H. He J. (2021b). Research status and development of key technologies for no-tillage seeding intellectualization. J. South China Agric. Univ. 42 (6), 27–35.

[B93] Wang S. Yang Z. (2020). The effect of the aging of agricultural labor force on the change of grain green total factor productivity. Res. Agric. Modern. 41 (3), 396–406. doi: 10.13872/j.1000-0275.220.0037

[B94] Wang Y. Yang J. Liang J. Qiang Y. Fang S. Gao M.. (2018). Analysis of the environmental behavior of farmers for non-point source pollution control and management in a water source protection area in China. Sci. Total Environ. 633, 1126–1135. doi: 10.1177/0954407012475272 29758864

[B95] Wang X. Yang H. Liu J. Wu J. Chen W. Wu J.. (2015). Effects of ditch-buried straw return on soil organic carbon and rice yields in a rice-wheat rotation system. Catena 127, 56–63. doi: 10.1016/j.catena.2014.10.012

[B96] Wang H. Zhang Y. Zhang Y. McDaniel M. Sun L. Su W.. (2020b). Water-saving irrigation is a ‘win-win’ management strategy in rice paddies – with both reduced greenhouse gas emissions and enhanced water use efficiency. Agric. Water Manage. 228, 105889. doi: 10.1016/j.agwat.2019.105889

[B97] Wu D. Wang Y. Qian W. (2020). Efficiency evaluation and dynamic evolution of china’s regional green economy: A method based on the super-PEBM model and DEA window analysis. J. Clean. Prod. 264, 121630. doi: 10.1016/j.jclepro.2020.121630

[B98] Wu P. Liu F. Li H. Cai T. Zhang P. Jia Z. (2021). ). suitable fertilizer application depth can increase nitrogen use efficiency and maize yield by reducing gaseous nitrogen losses. Sci. Total Environment. 781, 146787. doi: 10.1016/j.scitotenv.2021.146787

[B99] Xia H. Riaz M. Zhang M. Liu B. Li Y. El-Desouki Z.. (2022). Biochar-n fertilizer interaction increases n utilization efficiency by modifying soil C/N component under n fertilizer deep placement modes. Chemo. (Oxford). 286, 131594. doi: 10.1016/j.chemosphere.2021.131594 34346321

[B100] Xie R. Yuan Y. Huang J. (2017). Different types of environmental regulations and heterogeneous influence on “green” productivity: evidence from China. Ecol. Econ. 132, 104–112. doi: 10.1016/j.ecolecon.2016.10.019

[B101] Xie F. Zhang B. Wang N. (2021). Non-linear relationship between energy consumption transition and green total factor productivity: A perspective on different technology paths. Sustain. Prod. Consump. 28, 91–104. doi: 10.1016/j.spc.2021.03.036

[B102] Xu B. Chen W. Zhang G. Wang J. Ping W. Luo L.. (2020). How to achieve green growth in china’s agricultural sector. J. Clean. Prod. 271, 122770. doi: 10.1016/j.jclepro.2020.122770

[B103] Xue X. Gu X. (2022). Research on the threshold effect of non-grain on grain green total factor productivity. Chin. J. Agric. Resour. Reg. Plan. 43 (7), 17–26.

[B104] Yang X. Cheng L. Huang X. Zhang Y. Lebailly P. (2020). Incentive mechanism to promote corn stalk return sustainably in henan, china. Sci. Total Environ. 738, 139775. doi: 10.1016/j.scitotenv.2020.139775 32526418

[B105] Yang C. Hu P. Diao B. Cheng J. Cui H. (2021). Environmental performance evaluation of policies in main grain producing areas:from the perspective of agricultural carbon emissions. China Popul. Resour. Environ. 31 (12), 35–44.

[B106] Yang Y. Shi L. Zhang X. (2019). Application of mechanized deep tillage and subsoiling technology and types of machinery. Agric. Machin. Using Maintenance 8, 102. doi: 10.14031/j.cnki.njwx.2019.08.065

[B107] Yang H. Wang X. Bin P. (2022b). Agriculture carbon-emission reduction and changing factors behind agricultural eco-efficiency growth in China. J. Clean. Prod. 334, 130193. doi: 10.1016/j.jclepro.2021.130193

[B108] Yang Z. Wang D. Du T. Zhang A. Zhou Y. (2018). Total-factor energy efficiency in china’s agricultural sector: Trends, disparities and potentials. Energies (Basel) 11 (4), 853. doi: 10.3390/en11040853

[B109] Yang Z. Zhu Y. Zhang J. Li X. Ma P. Sun J.. (2022a). Comparison of energy use between fully mechanized and semi-mechanized rice production in southwest china. Energy 245, 123270. doi: 10.1016/j.energy.2022.123270

[B110] Yin C. Huang X. Zhao J. Cheng L. Chang Z. Chien H. (2016). Analysis of the willingness to accept for maize straw returned to field: based on farmer’s survey in hebei and shandong provinces. Chin. J. Agric. Resour. Regional Planning. 37 (7), 87–95.

[B111] Yu D. Liu L. Gao S. Yuan S. Shen Q. Chen H. (2022). Impact of carbon trading on agricultural green total factor productivity in China. J. Clean. Prod. 367, 132789. doi: 10.1016/j.jclepro.2022.132789

[B112] Zhai L. LÜ L. Dong Z. Zhang L. Zhang J. Jia X.. (2021). The water-saving potential of using micro-sprinkling irrigation for winter wheat production on the north China plain. J. Integr. Agri. 20 (6), 1687–1700. doi: 10.1016/S2095-3119(20)63326-3

[B113] Zhang W. Cao G. Li X. Zhang H. Wang C. Liu Q.. (2016). Closing yield gaps in China by empowering smallholder farmers. Nat. (London) 537 (7622), 671–674. doi: 10.1038/nature19368 27602513

[B114] Zhang Q. Chu Y. Xue Y. Ying H. Chen X. Zhao Y.. (2020). Outlook of china’s agriculture transforming from smallholder operation to sustainable production. Global Food Secur. 26, 100444. doi: 10.1016/j.gfs.2020.100444

[B115] Zhang M. Dong S. Zhu J. Zhao H. (2021). Research on the influence of socialized service of mid-production. J. Maize Sci. 06), 175–183. doi: 10.13597/j.cnki.maize.science.20210625

[B116] Zhang M. Song D. Pu X. Dang P. Qin X. Siddique K. (2022). Effect of different straw returning measures on resource use efficiency and spring maize yield under a plastic film mulch system. Eur. J. Agro. 134, 126461. doi: 10.1016/j.eja.2022.126461

[B117] Zhang C. Wu N. Zhang Y. Wu H. Gu F. Hu Z. (2018). Development status and trend of no tillage seeding technology at home and abroad. Jiangsu Agric. Sci. 46 (16), 1–5. doi: 10.15889/j.issn.1002-1302.2018.16.001

[B118] Zhao T. Xiao H. Zhao H. Dai J. Zhao L. Zhang M.. (2020). Response of maize yield, biological traits and nutrient utilization and soil fertility to fertilization depth in guizhou. J. Irrig. Drain. 39, 21–25. doi: 10.13522/j.cnki.ggps.2019113

[B119] Zhao Y. Xiong X. Wu C. (2021). Effects of deep placement of fertilizer on periphytic biofilm development and nitrogen cycling in paddy systems. Pedosphere 31 (1), 125–133. doi: 10.1016/S1002-0160(20)60051-0

[B120] Zhao P. Zeng L. Li P. Lu H. Hu H. Li C.. (2022). China’s transportation sector carbon dioxide emissions efficiency and its influencing factors based on the EBM DEA model with undesirable outputs and spatial durbin model. Energy 238, 121934. doi: 10.1016/j.energy.2021.121934

[B121] Zhao R. Liu Y. Tian M. Ding M. Cao L. Zhang Z.. (2018). Impacts of water and land resources exploitation on agricultural carbon emissions: The water-land-energy-carbon nexus. Land Use Policy 72, 480–492. doi: 10.1016/j.landusepol.2017.12.029

[B122] Zhong M. Huang G. He R. Lund H. Kaiser M. J. (2022). The technological innovation efficiency of china’s lithium-ion battery listed enterprises: evidence from a three-stage dea model and micro-data. Energy 246, 123331. doi: 10.1016/j.energy.2022.123331

[B123] Zhong X. Peng J. Kang X. Wu Y. Luo G. Hu W.. (2021). Optimizing agronomic traits and increasing economic returns of machine-transplanted rice with side-deep fertilization of double-cropping rice system in southern china. Field Crops Res. 270, 108191. doi: 10.1016/j.fcr.2021.108191

[B124] Zhuang Y. Zhang L. Li S. Liu H. Zhai L. Zhou F.. (2019). Effects and potential of water-saving irrigation for rice production in China. Agric. Water Manage. 217, 374–382. doi: 10.1016/j.agwat.2019.03.010

[B125] Zhu C. Ouyang Y. Diao Y. Yu J. Luo X. Zheng J.. (2021). Effects of mechanized deep placement of nitrogen fertilizer rate and type on rice yield and nitrogen use efficiency in chuanxi plain, China. J. Integr. Agri. 20 (2), 581–592. doi: 10.1016/S2095-3119(20)63456-6

[B126] Zou L. Liu Y. Wang Y. Hu X. (2020). Assessment and analysis of agricultural non-point source pollution loads in China: 1978–2017. J. Environ. Manage. 263, 110400. doi: 10.1016/j.jenvman.2020.110400 32174536

