# Supplementary material for: Threshold effects of green technology application on sustainable grain production: Evidence from China
Source: Front Plant Sci. 2023 Jan 30;14:1107970. doi: 10.3389/fpls.2023.1107970 (PMC9927014; doi:10.3389/fpls.2023.1107970)
Supplement: Supplementary file 1 [file Table_1.docx]

Supplementary Material

Threshold effects of green technology application on sustainable grain production: Evidence from China

Jingdong Li, Qingning Lin*

*** Correspondence:** Qingning Lin: linqingning@caas.cn

# Supplementary Tables

**TABLE S1** Application conditions, ecological effects and negative impacts of green technologies

| Name | Conditions | Ecological effects | Negative impacts |
| --- | --- | --- | --- |
| MDPS | Suitable soil texture, supporting machinery (tractor, rotary plough), reasonable farming time, skilled operation and experienced management (Yang et al., 2019). It is often used together with PSQS, MSRE and MFDD. | Improving soil, promoting crop root growth, increasing water use efficiency and organic carbon and nitrogen sequestration in soil, ect. (Shao et al., 2016; Wang et al., 2015; Wang et al., 2020a), and increasing crop production and reducing carbon footprint (Chen et al., 2020). | Excessive deep-plowing and subsoiling will change the soil structure, destroy the bottom layer of soil and reduce its water retention capacity (Baumhardt et al., 2008; Ding et al., 2018), thus reducing food production and damaging the natural environment. |
| PSQS | Good land consolidation, fertilization and irrigation conditions, complete agricultural machinery base, selection of improved varieties and reasonable control of planting structure. Enrichment seeding and prevention of pests and weeds are needed (CPGPRC, 2007). It is often combined with MDPS, MSRE, MFDD, etc. | Saving seeds, increasing survival rate of seedlings, increasing yield per unit area, stabilizing yield, and reducing weeding frequency and the use of herbicides (Karayel, 2009; Li et al.,2015; Jing et al., 2022). Low energy consumption and good ecological benefits (CPGPRC, 2007). | It has high requirements for production conditions, planting techniques and management (CPGPRC, 2007), and needs to be combined with other precision agriculture techniques, soil testing and formula fertilization techniques. In the context of small-scale agricultural production in China, inappropriate increase of the use of PSQS will not only raise production costs (Chen et al., 2022; Rahim et al., 2021) , but also bring more greenhouse gas emissions due to overuse of agricultural machinery. |
| MNTS | Complete agricultural machinery configuration, high-quality seeds, skilled operation and experienced management (Duan et al., 2022a). It is often combined with MSRE. | Improving carbon sequestration in soil and water and nitrogen use efficiency, reducing labor input and fuel consumption, resulting in significant environmental benefits and yield increase (Chaudhary et al., 2021; Keshavarz Afshar and Dekamin, 2022). | No-tillage sowing will consume more pesticide and cause serious non-point source pollution (Peixoto et al., 2020; Sun et al., 2022). Additionally, the constraints of seed quality, mechanical wear and the technical level of the operator may lead to decline in food production and waste of resources(Pisante et al., 2014; Wang et al., 2021b). |
| MSRE | Appropriate climate (high temperature and high precipitation), good agricultural machinery base, combined with land cover and appropriate crop rotation arrangements (Yin et al., 2016; Tian et al., 2019). It is applied together with MDPS, PSQS (or MNTS). | Preserving soil fertility, reducing fertilizer input, improving water conservation capacity of soil, increasing organic carbon content in soil, and alleviating resource pressure (Zhang et al., 2022; Sun et al., 2018). It is beneficial for improving crop yield and grain quality (Hu et al., 2022). | It has high production costs and high requirements for operation skills. Therefore, farmers’ willingness to adopt this technology is low (Wang et al., 2021a). Besides, excessive straw returning and improper technical operation hinders root penetration and slows down crop growth (Li et al., 2018). It can increase methane emissions and non-point source pollution (Hu et al., 2022), and lead to excessive heavy metal content in food (Yang et al., 2020). |
| MFDD | Complete agricultural machinery and equipment; selection of appropriate fertilizer deep application machine according to different crop varieties, fertilization stage and fertilization target; skilled operation, reasonable fertilizer and fertilization ratio, suitable fertilization depth and time (Zhao et al., 2020; Wang, 2021). | Reducing labor input, reducing fertilizer use and fertilizer loss, increasing fertility preservation time, and improving fertilizer use efficiency (Zhu et al., 2021; Zhong et al., 2021), reducing nitrification or denitrification of soil nitrogen (Wu et al., 2021), reducing greenhouse gas emissions (Gaihre et al., 2015), and effectively increasing crop yield (Miah et al., 2016). | It is highly dependent on agricultural machinery, and has high requirements for fertilizer ratio, fertilization time, operation technology and management in the process of fertilization. It is difficult for ordinary farmers to apply this technology. Improper use of this technology will lead to insufficient land fertility, waste of fertilizer applied, and decline in crop yield (Zhao et al., 2021; Xia et al., 2022). |
| WSIR | High agricultural mechanization base, supporting equipment maintenance and fertilization management (Chen et al., 2022), and skilled operation. Its application is limited by soil, climate and hydrological conditions and topography (Zhuang et al., 2019). | Improving water use efficiency (Man et al., 2014), reducing carbon emissions (Wang et al., 2020b), decreasing non-point source pollution, reducing pest risk, increasing crop yield (Zhuang et al., 2019), and improving environmental and economic benefits. | WSIR has high construction and maintenance cost of water-saving irrigation, as well as high requirement for operation technology, making it difficult for ordinary farmers to apply this technology (Zhuang et al., 2019). The decline of rural collective action in recent years is not conducive to the maintenance of water-saving irrigation facilities and the development of water-saving irrigation technology (Wang et al., 2022). |

**TABLE S2**  Calculation description of control variables

| Control variables | Calculation | Explanation |
| --- | --- | --- |
| Urbanization level (UR) | *UR_it_* = *UP_it_* / *TP_it_* | *UP_it_* is the urban population; *TP_it_* is the total population. |
| Agricultural mechanization level (AM) | *AM_it_* = *TAM_it_* / *TAP_it_* | *TAM_it_* is the total power of agricultural machinery; *TAP_it_* is the total agricultural planting area. |
| Irrigation level (IR) | *IR_it_* = *EIA_it_* / *TCA_it_* | *EIA_it_* is the effective irrigated area; *TCA_it_* is the total cultivated area. |
| Planting structure (PS) | *PS_it_* = [∑*_k_* (AG*_ikt_*×*GPA_ikt_*)] / [(∑*_k_* AG*_ikt_*)×(∑*_k_ GPA_ikt_*)] | AG*_ikt_* is the assignment of grain *k*; *GPA_ikt_* is the planting area of grain *k.* |
| Agricultural fiscal level (AF) | *AF_it_* = *LAF_it_* / *LFE_it_* | *LAF_it_* is the local agricultural fiscal expenditure; *LFE_it_* is the local fiscal expenditure. |
| Agricultural investment level (AI) | *AI_it_* = *FAA_it_* / *FAI_it_* | *FAA_it_* is the local fixed assets investment of agriculture; *FAI_it_* is the local fixed assets investment. |
| Rural income level (RI) | *RI_it_*= *PRR_it_* / *PUR_it_* | *PRR_it_* is the per capita income of rural residents; *PUR_it_* is the per capita income of urban residents. |
| Production agglomeration (PA) | *PA_it_* = ( *LGP_it_* / *LCP_it_*) / ( *NGP_t_* / *NCP_t_*) | *LGP_it_* is the local staple grain production; L*CP_it_* is the local crop production; *NGP_t_* is the national staple grain production; *NCP_t_* is the national crop production. |
| Trade dependence level (TD) | *TD_it_* = *APT_it_* / *GAO_it_* | *APT_it_* is the local agricultural products trade value; *GAO_it_* is the local gross agricultural output value |
| Disaster incidence level (DI) | *DI_it_*= *AD_it_* / *TAP_it_* | *AD_it_* is the agricultural disaster area; *TAP_it_* is the total agricultural planting area. |
| Temperature fluctuation level (TF) | *TF_it_* = *TE_it_* / *ATE_it_* | *TE_it_* is the local temperature; *ATE_it_* is the local average temperature in *t* years. |
| Precipitation fluctuation level (PF) | *PF_it_* = *PR_it_* / *APR_it_* | *PR_it_* is the local precipitation; *APR_it_* is the local average precipitation in *t* years. |

Note: *i* represents the province and *t* represents the year.

**TABLE S3**  Descriptive statistics of variables

| **Variable** | **Symbol** | **Units** | **Mean** | **Maximum** | **Minimum** | **Std.Dev.** |
| --- | --- | --- | --- | --- | --- | --- |
| Output variables for GTFPG calculation |  |  |  |  |  |  |
| Desirable output | — | 10^4^ t | 1788.1300 | 7022.5600 | 33.0600 | 1506.4190 |
| Undesirable output | — | 10^4^ t | 228.2290 | 777.4920 | 3.3708 | 167.5760 |
| Input variables for GTFPG calculation |  |  |  |  |  |  |
| Planting area | — | 10^4^ ha | 4678.7990 | 17024.4500 | 117.4500 | 3635.2120 |
| Fertilizer | — | 10^4^ t | 107.0487 | 409.4005 | 1.4792 | 87.1737 |
| Pesticide | — | 10^4^ t | 0.4959 | 2.4415 | 0.0022 | 0.5763 |
| Agricultural film | — | 10^4^ t | 1.3132 | 9.3556 | 0.0000 | 1.6975 |
| Diesel oil | — | 10^4^ t | 1.1179 | 10.0816 | 0.0009 | 1.4359 |
| Seed | — | 10^4^ t | 29.5552 | 141.7350 | 0.4418 | 31.0444 |
| Electricity for irrigation | — | 10^6^ kWh | 1660.1960 | 12863.7700 | 1.0223 | 2155.6700 |
| Labor | — | 10^6^ day | 350.5845 | 1510.5600 | 3.2574 | 251.5861 |
| Machinery | — | 10^6^ CNY | 4519.9260 | 30228.6200 | 30.7446 | 5236.2340 |
| Green technologies |  |  |  |  |  |  |
| Mechanical deep-plowing and subsoiling | MDPS | — | 0.2104 | 0.7874 | 0.0000 | 0.1570 |
| Precision and small quantity sown | PSQS | — | 0.1718 | 0.7833 | 0.0000 | 0.1945 |
| Mechanized no-tillage sown | MNTS | — | 0.0503 | 0.3777 | 0.0000 | 0.0766 |
| Mechanized straw returning | MSRE | — | 0.1685 | 0.7182 | 0.0000 | 0.1574 |
| Mechanical fertilizer deep distributing | MFDD | — | 0.2028 | 0.8238 | 0.0000 | 0.1928 |
| Water-saving irrigation | WSIR | — | 0.2198 | 0.8270 | 0.0214 | 0.1618 |
| Control variables |  |  |  |  |  |  |
| Urbanization level | UR | — | 0.1492 | 0.5236 | 0.0055 | 0.0867 |
| Agricultural mechanization level | AM | — | 5.6256 | 14.1562 | 1.3172 | 2.6269 |
| Irrigation level | IR | — | 0.4887 | 0.9551 | 0.1332 | 0.2098 |
| Planting structure | PS | — | 0.3436 | 0.5000 | 0.1951 | 0.1006 |
| Agricultural fiscal level | AF | — | 0.2556 | 1.7881 | 0.0199 | 0.2270 |
| Agricultural investment level | AI | — | 0.0374 | 0.1413 | 0.0019 | 0.0244 |
| Rural income level | RI | — | 0.3624 | 0.5466 | 0.2101 | 0.0663 |
| Production agglomeration | PA | — | 0.9246 | 1.5348 | 0.2533 | 0.2804 |
| Trade dependence level | TD | — | 0.2484 | 1.6188 | 0.0076 | 0.2852 |
| Disaster incidence level | DI | — | 0.2418 | 0.9359 | 0.0000 | 0.1610 |
| Temperature fluctuation level | TF | — | 1.0066 | 1.2912 | 0.7688 | 0.0591 |
| Precipitation fluctuation level | PF | — | 1.0008 | 1.6508 | 0.3876 | 0.2258 |

Note: “—” represent no data.

**TABLE S4** Threshold test results of GTFPG

| Region | Model | Threshold variables | Test forms | Threshold value-ℽ | F-value | 10% | 5% | 1% | Confidence interval-95% |
| --- | --- | --- | --- | --- | --- | --- | --- | --- | --- |
| Whole region | Model 1 | MDPS | Single-threshold | 0.029** | 14.891 | 11.433 | 12.794 | 16.453 | [0.019, 0.031] |
|  |  |  | Double-threshold | 0.296** | 13.870 | 9.431 | 10.600 | 14.085 | [0.290, 0.299] |
|  | Model 2 | PSQS | Single-threshold | 0.018** | 18.045 | 12.649 | 14.694 | 19.636 | [0.016, 0.021] |
|  |  |  | Double-threshold | 0.444** | 15.763 | 11.639 | 14.550 | 17.990 | [0.443, 0.447] |
|  | Model 3 | MNTS | Single-threshold | 0.027** | 14.162 | 9.742 | 11.096 | 17.927 | [0.025, 0.030] |
|  |  |  | Double-threshold | 0.133*** | 18.474 | 9.218 | 10.849 | 14.493 | [0.133, 0.134] |
|  | Model 4 | MSRE | Single-threshold | 0.087* | 14.937 | 13.113 | 15.316 | 19.311 | [0.084, 0.089] |
|  |  |  | Double-threshold | 0.209** | 14.322 | 10.116 | 11.649 | 14.895 | [0.203, 0.214] |
|  | Model 5 | MFDD | Single-threshold | 0.152* | 14.965 | 12.631 | 15.951 | 21.958 | [0.152, 0.155] |
|  |  |  | Double-threshold | 0.317* | 11.243 | 10.347 | 11.836 | 16.805 | [0.304, 0.322] |
|  | Model 6 | WSIR | Single-threshold | 0.161** | 14.799 | 11.214 | 13.431 | 16.861 | [0.157, 0.166] |
|  |  |  | Double-threshold | 0.395** | 13.270 | 8.755 | 10.819 | 13.374 | [0.372, 0.396] |
| Major grain producing areas | Model 7 | MDPS | Single-threshold | 0.091* | 14.302 | 13.236 | 15.170 | 20.022 | [0.086, 0.094] |
|  |  |  | Double-threshold | 0.289** | 13.367 | 10.545 | 11.832 | 19.354 | [0.286, 0.291] |
|  | Model 8 | PSQS | Single-threshold | 0.050* | 18.442 | 17.582 | 19.956 | 24.073 | [0.048, 0.052] |
|  |  |  | Double-threshold | 0.264** | 13.453 | 10.554 | 12.627 | 16.667 | [0.261, 0.267] |
|  | Model 9 | MNTS | Single-threshold | 0.172** | 15.773 | 13.311 | 14.794 | 23.747 | [0.170, 0.175] |
|  |  |  | Double-threshold | 0.339** | 15.432 | 11.676 | 13.253 | 16.174 | [0.317, 0.344] |
|  | Model 10 | MSRE | Single-threshold | 0.114** | 16.339 | 11.558 | 13.541 | 19.518 | [0.111, 0.117] |
|  |  |  | Double-threshold | 0.137 | 7.513 | 10.755 | 13.071 | 16.821 | [0.135, 0.140] |
|  | Model 11 | MFDD | Single-threshold | 0.462** | 16.127 | 12.515 | 15.746 | 20.922 | [0.445, 0.462] |
|  |  |  | Double-threshold | 0.496 | 7.041 | 10.525 | 12.421 | 17.303 | [0.491, 0.503] |
|  | Model 12 | WSIR | Single-threshold | 0.195** | 15.363 | 12.465 | 15.259 | 20.871 | [0.191, 0.197] |
|  |  |  | Double-threshold | 0.466*** | 17.602 | 10.934 | 12.192 | 16.674 | [0.263, 0.268] |
| Non-major grain producing areas | Model 13 | MDPS | Single-threshold | 0.068** | 18.286 | 12.147 | 15.671 | 23.235 | [0.061, 0.070] |
|  |  |  | Double-threshold | 0.141 | 6.215 | 9.879 | 11.798 | 15.492 | [0.129, 0.146] |
|  | Model 14 | PSQS | Single-threshold | 0.101* | 11.736 | 10.746 | 13.390 | 20.048 | [0.090, 0.106] |
|  |  |  | Double-threshold | 0.459* | 11.162 | 11.006 | 13.487 | 16.676 | [0.458, 0.476] |
|  | Model 15 | MNTS | Single-threshold | 0.055** | 15.660 | 10.732 | 12.486 | 16.807 | [0.054, 0.057] |
|  |  |  | Double-threshold | 0.250** | 13.482 | 11.053 | 12.927 | 17.101 | [0.245, 0.254] |
|  | Model 16 | MSRE | Single-threshold | 0.093** | 14.041 | 10.744 | 13.409 | 18.163 | [0.091, 0.094] |
|  |  |  | Double-threshold | 0.206* | 9.364 | 8.961 | 10.608 | 17.779 | [0.202, 0.209] |
|  | Model 17 | MFDD | Single-threshold | 0.036** | 17.373 | 12.031 | 14.846 | 18.801 | [0.035, 0.037] |
|  |  |  | Double-threshold | 0.295** | 16.092 | 11.841 | 13.631 | 17.468 | [0.291, 0.297] |
|  | Model 18 | WSIR | Single-threshold | 0.079** | 13.465 | 10.515 | 13.020 | 20.846 | [0.077, 0.081] |
|  |  |  | Double-threshold | 0.299** | 12.803 | 8.654 | 10.728 | 14.920 | [0.294, 0.301] |

Note: ‘*’, ‘**’, ‘***’ represent the significance levels of 10%, 5% and 1%, respectively.

**TABLE S5** Threshold test results of GECG

| Region | Model | Threshold variables | Test forms | Threshold value-ℽ | F-value | 10% | 5% | 1% | Confidence interval-95% |
| --- | --- | --- | --- | --- | --- | --- | --- | --- | --- |
| Whole region | Model 19 | MDPS | Single-threshold | 0.067* | 15.863 | 13.850 | 16.267 | 22.041 | [0.065, 0.068] |
|  |  |  | Double-threshold | 0.346** | 14.472 | 10.283 | 11.779 | 16.963 | [0.337, 0.351] |
|  | Model 20 | PSQS | Single-threshold | 0.120* | 13.492 | 12.858 | 14.790 | 20.625 | [0.115, 0.124] |
|  |  |  | Double-threshold | 0.301 | 6.319 | 10.368 | 11.772 | 16.774 | [0.292, 0.307] |
|  | Model 21 | MNTS | Single-threshold | 0.096** | 16.457 | 11.804 | 11.560 | 17.452 | [0.091, 0.102] |
|  |  |  | Double-threshold | 0.231 | 3.314 | 11.560 | 13.857 | 19.138 | [0.227, 0.236] |
|  | Model 22 | MSRE | Single-threshold | 0.109* | 16.975 | 12.921 | 17.271 | 24.021 | [0.107, 0.114] |
|  |  |  | Double-threshold | 0.386* | 14.323 | 13.044 | 15.672 | 23.772 | [0.383, 0.388] |
|  | Model 23 | MFDD | Single-threshold | 0.032** | 14.276 | 11.762 | 14.168 | 17.968 | [0.029, 0.032] |
|  |  |  | Double-threshold | 0.122* | 12.492 | 11.514 | 13.757 | 20.639 | [0.119, 0.130] |
|  | Model 24 | WSIR | Single-threshold | 0.067** | 17.123 | 12.114 | 13.749 | 20.091 | [0.065, 0.069] |
|  |  |  | Double-threshold | 0.146*** | 17.465 | 9.875 | 12.687 | 17.064 | [0.142, 0.148] |
| Major grain producing areas | Model 25 | MDPS | Single-threshold | 0.117*** | 16.185 | 8.626 | 10.426 | 13.172 | [0.117, 0.118] |
|  |  |  | Double-threshold | 0.375** | 16.773 | 10.957 | 12.834 | 17.311 | [0.374, 0.380] |
|  | Model 26 | PSQS | Single-threshold | 0.103*** | 15.729 | 11.716 | 13.313 | 15.403 | [0.100,0.107] |
|  |  |  | Double-threshold | 0.305 | 7.724 | 13.705 | 15.256 | 18.833 | [0.298, 0.320] |
|  | Model 27 | MNTS | Single-threshold | 0.085* | 8.634 | 8.509 | 10.572 | 14.553 | [0.078, 0.086] |
|  |  |  | Double-threshold | 0.101 | 5.185 | 8.984 | 10.988 | 16.318 | [0.100, 0.102] |
|  | Model 28 | MSRE | Single-threshold | 0.121** | 10.357 | 7.222 | 9.014 | 11.547 | [0.120, 0.126] |
|  |  |  | Double-threshold | 0.513*** | 15.263 | 6.479 | 7.518 | 9.762 | [0.505, 0.519] |
|  | Model 29 | MFDD | Single-threshold | 0.152** | 14.701 | 10.803 | 13.889 | 20.714 | [0.149, 0.154] |
|  |  |  | Double-threshold | 0.425* | 13.411 | 11.702 | 17.281 | 22.406 | [0.423, 0.425] |
|  | Model 30 | WSIR | Single-threshold | 0.046** | 13.629 | 6.768 | 9.044 | 19.844 | [0.044, 0.047] |
|  |  |  | Double-threshold | 0.194*** | 13.865 | 6.507 | 7.674 | 10.605 | [0.194, 0.199] |
| Non-major grain producing areas | Model 31 | MDPS | Single-threshold | 0.122** | 10.490 | 8.753 | 10.304 | 13.445 | [0.092, 0.131] |
|  |  |  | Double-threshold | 0.414** | 11.792 | 7.829 | 10.741 | 15.072 | [0.412, 0.419] |
|  | Model 32 | PSQS | Single-threshold | 0.106** | 10.791 | 8.170 | 9.585 | 12.996 | [0.094, 0.118] |
|  |  |  | Double-threshold | 0.274** | 12.684 | 9.661 | 10.928 | 14.698 | [0.271, 0.280] |
|  | Model 33 | MNTS | Single-threshold | 0.123** | 10.423 | 8.235 | 9.718 | 14.295 | [0.120, 0.125] |
|  |  |  | Double-threshold | 0.229 | 4.876 | 8.512 | 11.012 | 18.204 | [0.218, 0.232] |
|  | Model 34 | MSRE | Single-threshold | 0.086* | 8.429 | 7.692 | 9.556 | 13.739 | [0.082, 0.088] |
|  |  |  | Double-threshold | 0.142*** | 13.641 | 7.937 | 9.741 | 11.822 | [0.140, 0.143] |
|  | Model 35 | MFDD | Single-threshold | 0.122** | 11.718 | 9.576 | 11.334 | 14.711 | [0.117, 0.123] |
|  |  |  | Double-threshold | 0.263 | 10.533 | 13.111 | 14.839 | 18.885 | [0.260, 0.269] |
|  | Model 36 | WSIR | Single-threshold | 0.067 | 8.042 | 8.279 | 9.929 | 12.972 | [0.065, 0.069] |
|  |  |  | Double-threshold | 0.144** | 9.768 | 7.281 | 8.297 | 10.046 | [0.136, 0.146] |

Note: ‘*’, ‘**’, ‘***’ represent the significance levels of 10%, 5% and 1%, respectively.

**TABLE S6** Threshold test results of GTCG

| Region | Model | Threshold variables | Test forms | Threshold value-ℽ | F-value | 10% | 5% | 1% | Confidence interval-95% |
| --- | --- | --- | --- | --- | --- | --- | --- | --- | --- |
| Whole region | Model 37 | MDPS | Single-threshold | 0.095* | 16.061 | 14.735 | 17.757 | 21.861 | [0.094, 0.101] |
|  |  |  | Double-threshold | 0.229 | 7.882 | 9.983 | 12.089 | 15.693 | [0.224, 0.231] |
|  | Model 38 | PSQS | Single-threshold | 0.003 | 6.351 | 13.946 | 17.019 | 22.584 | [0.003, 0.004] |
|  |  |  | Double-threshold | 0.080 | 6.457 | 11.447 | 13.271 | 21.581 | [0.072, 0.089] |
|  | Model 39 | MNTS | Single-threshold | 0.081* | 15.142 | 13.014 | 15.893 | 22.143 | [0.080, 0.083] |
|  |  |  | Double-threshold | 0.152 | 9.429 | 10.120 | 11.510 | 15.881 | [0.147, 0.155] |
|  | Model 40 | MSRE | Single-threshold | 0.025* | 15.633 | 14.382 | 16.505 | 24.205 | [0.021, 0.027] |
|  |  |  | Double-threshold | 0.156 | 9.942 | 10.676 | 13.339 | 25.774 | [0.149, 0.157] |
|  | Model 41 | MFDD | Single-threshold | 0.064** | 15.054 | 12.359 | 14.189 | 18.604 | [0.061, 0.071] |
|  |  |  | Double-threshold | 0.377*** | 15.173 | 9.549 | 11.049 | 14.093 | [0.371, 0.383] |
|  | Model 42 | WSIR | Single-threshold | 0.141** | 13.221 | 10.694 | 11.888 | 16.137 | [0.140, 0.144] |
|  |  |  | Double-threshold | 0.155 | 5.40 | 8.166 | 9.931 | 12.867 | [0.154, 0.159] |
| Major grain producing areas | Model 43 | MDPS | Single-threshold | 0.117** | 11.816 | 8.125 | 9.004 | 12.018 | [0.113, 0.119] |
|  |  |  | Double-threshold | 0.186 | 9.019 | 9.649 | 10.885 | 16.674 | [0.183, 0.192] |
|  | Model 44 | PSQS | Single-threshold | 0.103*** | 12.135 | 9.112 | 10.393 | 11.977 | [0.100, 0.107] |
|  |  |  | Double-threshold | 0.444** | 17.481 | 10.537 | 16.893 | 23.279 | [0.437, 0.445] |
|  | Model 45 | MNTS | Single-threshold | 0.090** | 11.248 | 9.158 | 10.497 | 14.216 | [0.088, 0.092] |
|  |  |  | Double-threshold | 0.145* | 10.425 | 9.191 | 10.795 | 13.614 | [0.144, 0.146] |
|  | Model 46 | MSRE | Single-threshold | 0.037* | 6.788 | 6.527 | 7.746 | 9.105 | [0.033, 0.039] |
|  |  |  | Double-threshold | 0.352** | 11.520 | 7.845 | 8.986 | 11.847 | [0.315, 0.355] |
|  | Model 47 | MFDD | Single-threshold | 0.049** | 12.227 | 9.191 | 10.441 | 12.332 | [0.046, 0.052] |
|  |  |  | Double-threshold | 0.371* | 14.953 | 10.907 | 14.792 | 17.664 | [0.368, 0.383] |
|  | Model 48 | WSIR | Single-threshold | 0.093*** | 9.779 | 7.006 | 7.839 | 9.669 | [0.091,0.099] |
|  |  |  | Double-threshold | 0.176*** | 14.001 | 7.150 | 8.135 | 11.018 | [0.173, 0.179] |
| Non-major grain producing areas | Model 49 | MDPS | Single-threshold | 0.178** | 9.489 | 7.541 | 8.638 | 10.739 | [0.176, 0.182] |
|  |  |  | Double-threshold | 0.206 | 6.635 | 8.187 | 10.666 | 15.966 | [0.201, 0.209] |
|  | Model 50 | PSQS | Single-threshold | 0.104* | 9.160 | 8.159 | 9.924 | 13.844 | [0.101, 0.106] |
|  |  |  | Double-threshold | 0.273*** | 15.092 | 8.433 | 9.393 | 10.658 | [0.270, 0.280] |
|  | Model 51 | MNTS | Single-threshold | 0.182* | 11.608 | 11.362 | 14.256 | 18.597 | [0.174, 0.192] |
|  |  |  | Double-threshold | 0.211 | 7.282 | 9.474 | 11.181 | 14.483 | [0.207, 0.213] |
|  | Model 52 | MSRE | Single-threshold | 0.134** | 11.942 | 7.679 | 9.131 | 13.390 | [0.132, 0.135] |
|  |  |  | Double-threshold | 0.176 | 7.602 | 8.821 | 10.561 | 13.422 | [0.175, 0.179] |
|  | Model 53 | MFDD | Single-threshold | 0.036** | 10.496 | 8.093 | 9.883 | 11.896 | [0.035, 0.039] |
|  |  |  | Double-threshold | 0.291** | 11.556 | 9.398 | 10.913 | 15.346 | [0.290, 0.295] |
|  | Model 54 | WSIR | Single-threshold | 0.143** | 10.526 | 6.737 | 7.692 | 11.066 | [0.141, 0.146] |
|  |  |  | Double-threshold | 0.180 | 4.485 | 7.087 | 7.881 | 9.341 | [0.174, 0.187] |

Note: ‘*’, ‘**’, ‘***’ represent the significance levels of 10%, 5% and 1%, respectively.
